# A Downstream CpG Island Controls Transcript Initiation and Elongation and the Methylation State of the Imprinted *Airn* Macro ncRNA Promoter

**DOI:** 10.1371/journal.pgen.1002540

**Published:** 2012-03-01

**Authors:** Martha V. Koerner, Florian M. Pauler, Quanah J. Hudson, Federica Santoro, Anna Sawicka, Philipp M. Guenzl, Stefan H. Stricker, Yvonne M. Schichl, Paulina A. Latos, Ruth M. Klement, Katarzyna E. Warczok, Jacek Wojciechowski, Christian Seiser, Robert Kralovics, Denise P. Barlow

**Affiliations:** 1 CeMM–Research Center for Molecular Medicine of the Austrian Academy of Sciences, Vienna, Austria; 2 Max F. Perutz Laboratories, Department of Medical Biochemistry, Medical University of Vienna, Vienna, Austria; 3 IMP/IMBA Transgenic Service, Research Institute of Molecular Pathology, Vienna, Austria; Medical Research Council Human Genetics Unit, United Kingdom

## Abstract

A CpG island (CGI) lies at the 5′ end of the *Airn* macro non-protein-coding (nc) RNA that represses the flanking *Igf2r* promoter *in cis* on paternally inherited chromosomes. In addition to being modified on maternally inherited chromosomes by a DNA methylation imprint, the *Airn* CGI shows two unusual organization features: its position immediately downstream of the *Airn* promoter and transcription start site and a series of tandem direct repeats (TDRs) occupying its second half. The physical separation of the *Airn* promoter from the CGI provides a model to investigate if the CGI plays distinct transcriptional and epigenetic roles. We used homologous recombination to generate embryonic stem cells carrying deletions at the endogenous locus of the entire CGI or just the TDRs. The deleted *Airn* alleles were analyzed by using an ES cell imprinting model that recapitulates the onset of *Igf2r* imprinted expression in embryonic development or by using knock-out mice. The results show that the CGI is required for efficient *Airn* initiation and to maintain the unmethylated state of the *Airn* promoter, which are both necessary for *Igf2r* repression on the paternal chromosome. The TDRs occupying the second half of the CGI play a minor role in *Airn* transcriptional elongation or processivity, but are essential for methylation on the maternal *Airn* promoter that is necessary for *Igf2r* to be expressed from this chromosome. Together the data indicate the existence of a class of regulatory CGIs in the mammalian genome that act downstream of the promoter and transcription start.

## Author Summary

CpG islands are CG-rich regions associated with the majority of mammalian promoters. Although widely considered to be necessary for promoter activity, their exact function is unknown. CpG islands are mostly unmethylated during development and differentiate with notable exceptions including imprinted genes and genes on the inactive X chromosome. Here we analysed how the imprinted *Airn* ncRNA CpG island, that is normally methylated on the maternal and unmethylated on the paternal chromosome, regulates its associated upstream promoter. We used embryonic stem cells or mice carrying a deletion of either the whole CpG island or of the second half that contains an unusual series of tandem direct repeats. Our results show that the CpG island is needed for efficient transcription of the *Airn* promoter on the paternal chromosome and to keep it free from DNA methylation. The series of tandem direct repeats plays a minor role in regulating the length of the *Airn* transcript on the paternal chromosome but is essential for DNA methylation of the *Airn* promoter on the maternal chromosome. These results show that CpG islands do not only function as classical promoters to bind RNA polymerase II and initiate transcription, but can also play other roles in regulating transcription processivity as well as the epigenetic state of their associated promoter.

## Introduction

Atypical CpG-rich regions known as CpG islands (CGIs) overlap 60–70% of mammalian transcription start sites [1]. Although most CGIs extend downstream of the transcription start and are therefore partly transcribed, they are considered to have promoter regulatory functions and are often described as ‘CGI promoters’. A recent study used a biochemical purification strategy to identify a large number of novel CGIs not associated with annotated promoters, in the body of coding genes or in intergenic regions [2]. While this could indicate the mammalian genome has many transcripts still to be identified, it is also possible that CGIs have additional functions in addition to promoter regulation.

The best examples of CGIs with additional regulatory functions are those that lie inside imprint control elements (ICE, also known as an imprint control region) [3]. An ICE is a genetically defined region whose epigenetic state controls parental-specific expression of small clusters of genes [4]–[6]. CGIs within an ICE are similar to classic promoter-associated CGIs as they show a CpG density higher than the genome average and lack sequence conservation, even between homologous mouse and human elements [7]. However they differ in several ways [8], [9]. First, their CpG density is less than that of classic promoter-associated CGIs. Second, whereas most promoter-associated CGIs are free of DNA methylation [1], CGIs within ICEs gain DNA methylation during gametogenesis but only in one of the two parental gametes. These modified regions are also known as gametic ‘differentially-methylated-regions’ (gDMRs), since once established in a gamete they are maintained in all somatic cells on the same parental chromosome, while the other parental allele remains methylation-free. In six imprinted clusters the ICE has been shown by deletion experiments that include the CGI, to control repression of all imprinted genes [10]–[15]. Thus, the third distinguishing feature is that an unmethylated ICE can act as a *cis*-acting long-range repressor of multiple flanking genes. This indicates that CGIs residing in an ICE may be a prototype for a class of *cis*-regulatory CGIs that may differ from classic promoter-associated CGIs.

Remarkably, the silencing ability of the unmethylated ICE correlates with its action as a promoter or *cis*-activator of a macro non-protein-coding (nc) RNA (provisionally defined as a ncRNA >200 bp whose function does not depend on processing to smaller RNAs) [16], [17]. Three imprinted macro ncRNAs that play a direct role in imprinted gene silencing i.e., *Airn*, *Kcnq1ot1*, and *Nespas*, have their promoter in the ICE [18]–[20]. These *cis*-repressor macro ncRNAs therefore contain CGI sequences at their 5′ end that could contribute to their repressor function. The *Airn*, *Kcnq1ot1* and *Nespas* macro ncRNAs are expressed only from the paternal chromosome and induce paternal-specific silencing of flanking protein-coding genes. Imprinted expression of the flanking protein-coding genes arises because these repressor macro ncRNAs are repressed on the maternal chromosome by an ICE gametic methylation imprint [9], [21], [22]. Maternal gametic methylation imprints depend on expression in growing oocytes of the DNMT3A/B *de novo* methyltransferases and the DNMT3L cofactor [23], [24]. It has also been shown that transcription across the ICE controlling *Nespas* ncRNA expression is required for methylation in oocytes [25]. In addition, recent high-throughput analyses show a general link between overlapping transcription and CGI methylation in oocytes [26]. However, there is little information on the relative contribution of DNA elements within the ICE for the methylation state. Tandem direct repeats (TDRs) that show organizational but not sequence conservation, are frequently found in or adjacent to the ICE and have been suggested to guide epigenetic modifications [9], [27], [28]. The TDRs are present on both parental chromosomes but methylation of the ICE restricts expression of the macro ncRNA to one parental chromosome. Thus, it is possible that the TDRs play a role in ICE methylation on one parental chromosome and in the repressor function of the macro ncRNA expressed from the other parental chromosome. However, to date various experiments analysing their function either at the endogenous locus or in a transgene context, have not yet identified a general function for TDRs in imprinted clusters [28].

**Figure 1:**
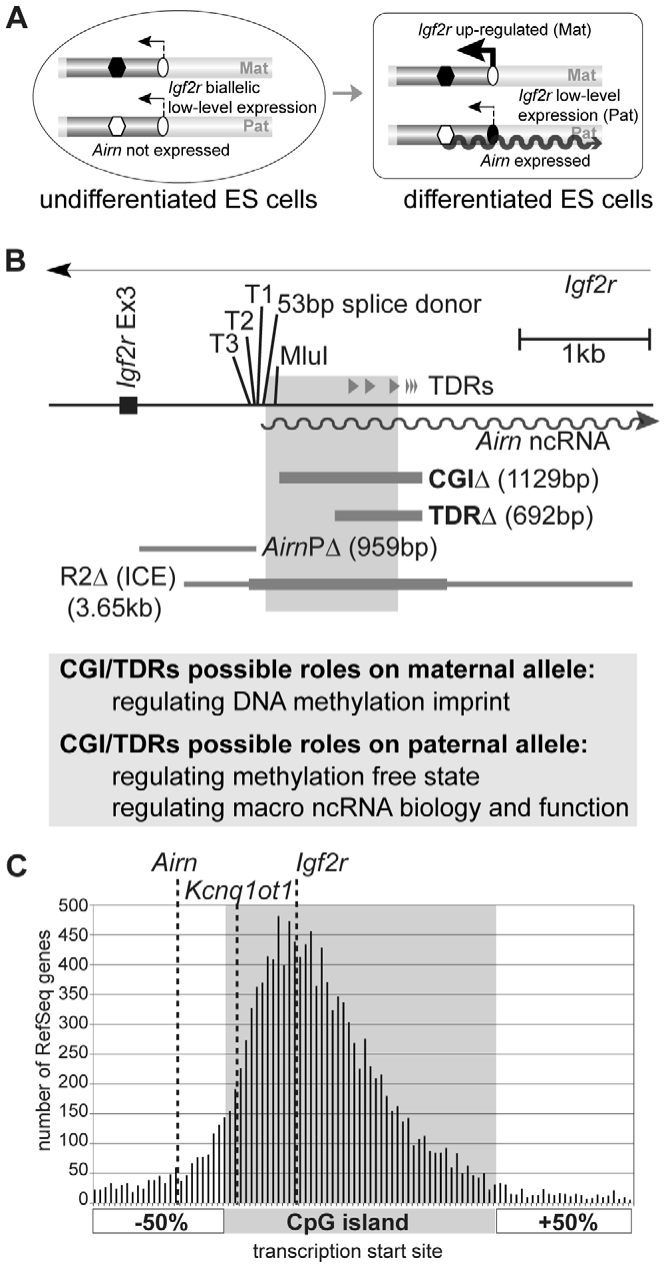
CpG islands lie upstream and downstream of the transcription start site. (A) ES cell imprinting model. In undifferentiated ES cells *Igf2r* is expressed from both chromosomes and *Airn* is not expressed [31]. The ICE, which contains the *Airn* promoter, is methylated on the maternal allele (black hexagon) and unmethylated on the paternal allele (white hexagon). The *Igf2r* promoter is unmethylated on both parental alleles (white oval). During differentiation, *Airn* (wavy line) is expressed from the unmethylated paternal allele. *Igf2r* is upregulated up to 20-fold on the maternal allele, but its upregulation on the paternal allele is blocked by *Airn* expression *in cis*. The repressed paternal *Igf2r* promoter gains DNA methylation late in differentiation (black oval). (B) *Airn* 5′ region. T1,T2,T3: multiple closely-spaced *Airn* TSSs. 53 bp splice donor: shared by all *Airn* splice variants. MluI: MluI restriction site. Grey shading: CGI. Grey triangles labelled TDRs: tandem direct repeats. *CGIΔ*/*TDRΔ*: 1129/692 bp deletions generated here. *AirnPΔ*: 959 bp deletion defining *Airn* promoter [32] that lies upstream of the CGI. *R2Δ*: 3656 bp deletion defining the imprint control element or ICE [10]. Thicker grey line on *R2Δ*: maternally methylated region. Possible roles of the CGI and the TDRs on the two parental alleles are listed below. (C) Transcription start sites (TSSs) of mouse RefSeq genes are plotted relative to their CpG island (CGI) and 50% of the CGI length upstream and downstream (see Materials and Methods). Dotted line: TSS of *Airn*, *Kcnq1ot1* and *Igf2r*. The *Airn* TSS lies upstream to the CGI in contrast to the majority of TSSs that lie inside of the CGI.

The *Airn* macro ncRNA promoter that is embedded in the ICE, lies in intron 2 of the *Igf2r* gene. *Airn* overlaps and represses the *Igf2r* promoter (Figure 1A); in extra-embryonic lineages *Airn* also represses the non-overlapped *Slc22a2* and *Slc22a3* genes that lie more than 100 kb upstream of the *Airn* promoter [19], [29]. *Airn* is an unusually long 118 kb ncRNA that is transcribed by RNA polymerase II (RNAPII). The majority of nascent *Airn* transcripts are unspliced and nuclear-localized while the minority that are spliced are exported to the cytoplasm [21]. Splicing suppression, unusual length and gene silencing ability are also features shared with the *Kcnq1ot1* macro ncRNA [30]. Previously we have established an ES cell imprinting model that recapitulates the onset of imprinted *Igf2r* expression in early mouse embryonic development [31] (Figure 1A). Undifferentiated ES cells show bi-allelic but low-level *Igf2r* expression and *Airn* is not expressed [31]. *Airn* expression is initiated during ES cell differentiation and induces imprinted *Igf2r* expression by blocking up-regulation of the overlapped paternal promoter between days 3–5. The *Igf2r* promoter, which is also associated with a CGI, gains DNA methylation on the paternal allele after the onset of imprinted expression between days 5–14. However, this somatic methylation mark is not required for repression, as *Airn* silences *Igf2r* in mouse embryos lacking DNA methylation [21], [22]. We previously used a deletion/replacement approach in this ES cell imprinting model to identify a 959 bp promoter region immediately upstream of the *Airn* main transcription start [32]. These experiments demonstrated not only that the promoter lies upstream of the annotated CGI (Figure 1B), but also, that the endogenous *Airn* promoter does not control the unusual features of the macro ncRNA, as *Airn* driven by the mouse *Pgk1* promoter is indistinguishable from *Airn* driven by its endogenous promoter [32]. Thus control of the unusual biology of the *Airn* macro ncRNA lies outside its promoter.

Here we use the ES cell imprinting model and also mouse models, to test if the *Airn* downstream CGI plays a role either on the paternal allele in regulating *Airn* expression and function and the unmethylated state of the ICE or, on the maternal allele in regulating ICE methylation (Figure 1A, 1B). The *Airn* downstream CGI contains in its distal half two classes of imperfect TDRs that are each repeated three times, one with a 172–180 bp monomer length and one with a 30–32 bp monomer length (Figure 1B). We used homologous recombination in ES cells to delete a 1129 bp fragment containing the entire CGI and also to delete a 692 bp fragment containing just the TDRs. Both deletions left the *Airn* promoter and transcription start site (TSS) intact. Analysis of the effects of the deletion on the paternal chromosome that expresses *Airn* shows that the CGI deletion decreased *Airn* transcription initiation and strongly reduced transcript elongation, which as predicted from previous analyses [19], led to a loss of its ability to repress *Igf2r in cis*. The TDR deletion on the paternal chromosome led to a minor defect in transcript processivity that progressively affected the 3′ end of *Airn*, combined with a minor effect on its repressor function in differentiated ES cells and in mouse tissues. In contrast to the minor role on the paternal chromosome, analysis in mouse embryos of maternal chromosomes carrying the TDR deleted allele shows this element is essential for the DNA methylation imprint. Together these data show the *Airn* CGI has a dual parental-specific function and is necessary both for *Airn* biology and function as well as the critical epigenetic modifications that control its imprinted expression.

## Results

### CGIs can lie up- and downstream of the TSS

We first examined if the position of the *Airn* CGI that lies downstream of the TSS, represents a rare exception or a common occurrence in the mouse genome (Figure 1C). We asked, for each CGI annotated by the UCSC genome browser (http://genome.ucsc.edu/), if a known protein-coding or non-protein-coding gene taken from the NCBI RNA reference sequences collection (RefSeq), has its TSS within the CGI or in the DNA region representing half the length of the CGI up- or downstream. 57% of all RefSeq genes were associated with a CGI, of which 88.5% including *Igf2r*, have their TSSs within the body of the CGI (grey shaded area Figure 1C), with the majority lying in the first half. 11.5% of CGI-associated RefSeq genes have their TSS located outside of the annotated CGI, 8.7% of these have their TSS upstream and 2.8% have a TSS downstream of the CGI. *Airn* represents one of those with their TSS located upstream of the CGI while the *Kcnq1ot1* macro ncRNA has its TSS on the 5′ border of the CGI. As the *Airn* macro ncRNA has its TSS located upstream of the CGI it is possible to distinguish separate functions for the promoter (previously mapped to a 959 bp fragment lying upstream of the *Airn*-TSS [32]) and the CGI.

### Generation of TDR deletion embryonic stem (ES) cells

We first generated a deletion of the TDRs that occupy the second half of the *Airn* downstream CGI (Figure 1B). We used feeder-dependent D3 ES cells previously modified to contain a single nucleotide polymorphism (SNP) in *Igf2r* exon 12 that is used to distinguish maternal and paternal *Igf2r* expression [31]. The SNP modifies the maternal allele, thus this cell line is called *S12/+* (note the maternal allele is always written on the left *i.e.*, Mat/Pat). *S12/+* cells were used as the wildtype control for all differentiation experiments. We targeted *S12/+* cells by homologous recombination to delete a 692 bp region containing all TDRs starting 614 bp downstream of the major *Airn*-TSS (T1). The selection cassette was inserted 2 kb upstream of the deletion to avoid leaving a loxP site at the deletion, which has been reported to attract DNA methylation [33] and also to minimise potential effects on the *Airn* promoter region from the transient presence of a selection cassette (Figure S1A). Two independent homologously-targeted clones (named *S12/TDRΔ+cas*-1 and -2) were verified by Southern blot. The selection cassette was removed by transient transfection with a CRE-recombinase expressing plasmid (Figure S1A, S1B) and cells were subcloned to obtain four cell lines (named *S12/TDRΔ*-1A/-1B/-2A/-2B). Southern blot analysis showed that all cells were targeted on the paternal allele that carries the unmethylated active ICE and expresses the *Airn* ncRNA (Figure S1C, S1D). Preferential paternal targeting of the region between the *Airn* and *Igf2r* promoters is a feature of this cluster (data not shown). Initial analysis of the deletion was performed in the ES cell imprinting model that we have shown recapitulates the onset of imprinted *Igf2r* expression in the early embryo [31], [32]. To further validate the ES cell imprinting model and to observe a possible role of the TDRs in ICE methylation on the maternal allele, we deleted the same region in intraspecies 129/B6 A9-ES cells to generate knock-out mice (Figure S2A). The homologously-targeted A9 clone (*+/TDRΔ+cas*) was injected into blastocysts and the selection cassette removed by mating to a MORE CRE-deleter strain (Figure S2B, S2C) [34].

### The TDRs play a role in *Airn* transcript processivity

**Figure 2:**
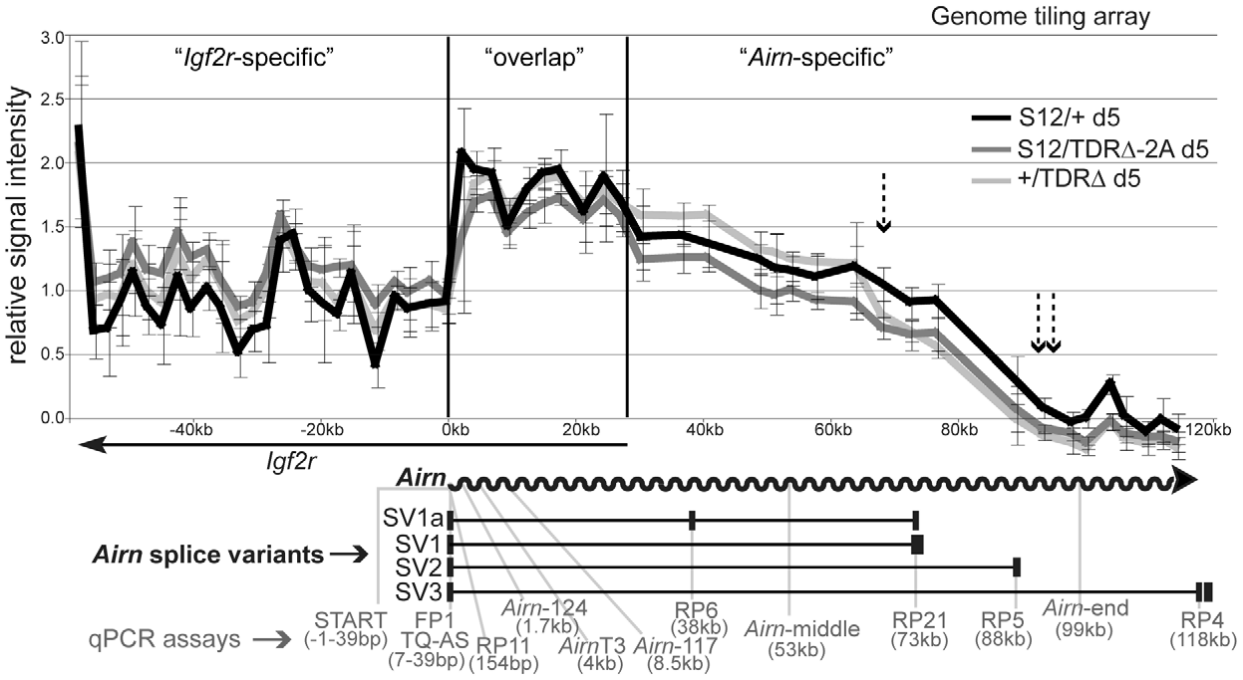
Tandem direct repeats play a role in *Airn* processivity. *Airn* expression by genome tiling array in day 5 differentiated ES cells carrying a paternal wildtype (*S12/+*) or mutated (*S12/TDRΔ*-2A and *+/TDRΔ*) allele. Note the maternal allele is always written on the left side (Mat/Pat). x-axis: basepairs, y-axis: averaged relative signal intensities with standard deviation (see Materials and Methods). Single and double dashed arrows: position after which consistent differences between wildtype and two *TDRΔ* cell lines are seen. Grey arrow: *Airn* hybridisation signals are lost after 90 kb in two *TDRΔ* cell lines. Below: *Airn* (wavy arrow) and *Airn* splice variants (black boxes: exons). Grey font: *Airn* qPCR assays with their distance from *Airn*-TSS. RP11, RP6, RP21, RP5, RP4 were combined with FP1+TQ-AS. This analysis shows that *Airn* in *TDRΔ* cells is reduced after 68 kb and lost after 90 kb.

It is technically challenging to determine the exact length of macro ncRNAs as they are too long to be resolved on RNA blots. Therefore to test if the TDR deletion changed the length of the *Airn* transcript, we performed an RNA hybridisation to a genome tiling array. Genome tiling arrays allow the detection of changes in transcript length using approximately 50 bp long probes spaced every 100 bp of single copy genomic DNA. As *Airn* is only expressed upon differentiation, we differentiated wildtype (*S12/+*) and two independently derived *TDRΔ* ES cells (*S12/TDRΔ*-2A and *+/TDRΔ*). cDNA prepared from total RNA labelled with one fluorochrome and sonicated genomic DNA labelled with a second fluorochrome, were cohybridized to the tiling array and the relative signal intensity plotted (Figure 2). The displayed 180 kb region contains the overlapping *Igf2r* and *Airn* transcripts and can be divided into three parts: the first part is specific to the 3′ end of the *Igf2r* transcript, the second part is the region of *Igf2r*/*Airn* sense/antisense transcriptional overlap and the third part is specific to the 3′ end of the *Airn* transcript from 28–118 kb. In the ‘*Igf2r*-specific’ region, comparison of signal intensities does not indicate a difference in *Igf2r* levels between the wildtype and two *TDRΔ* ES cell lines, as the overlapping error bars show the technical variation is larger than the biological difference. All three relative signal intensities then show an abrupt increase in the ‘overlap’ region due to the combined signals of *Igf2r* and *Airn*. In the ‘*Airn*-specific’ region, all three signal intensities decline from left to right as previously shown for wildtype *Airn* [32]. However, in both *TDRΔ* cell lines compared to wildtype cells, although *Airn* relative intensity was unchanged from 28–68 kb downstream of the *Airn*-TSS, it was reduced after 68 kb and absent from 90 kb onwards while wildtype *Airn* extends 20 kb further (Figure 2 single and double dashed arrows). Thus the TDR deletion on the paternal chromosome has no effect on the first half of *Airn* but reduces its overall length progressively towards the 3′ end.

**Figure 3:**
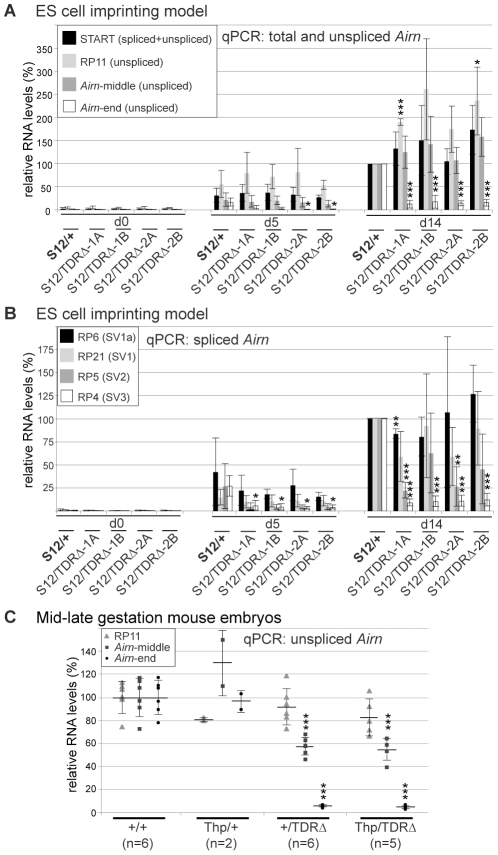
Tandem direct repeats regulate the length of *Airn*. (A) qPCR of total (spliced+unspliced) *Airn* in *S12/+* and four *S12/TDRΔ* cell lines (1A/1B/2A/2B), in undifferentiated (d0) and day 5 or 14 differentiated ES cells (see Figure 2 map for location of qPCR assays). Relative *Airn* levels were set to 100% in *S12/+* cells at d14. Bars and error bars: mean and standard deviation of three differentiation sets. *S12/+* and *S12/TDRΔ* were compared using an unpaired t-test (*P = 0.1–0.5, **P = 0.001–0.01, ***P<0.001). The data show that *Airn* steady-state levels are unchanged up to 53 kb but are greatly reduced and lost at the 3′ end. (B) qPCR of spliced *Airn* in *S12/+* and four *S12/TDRΔ* cell lines (1A/1B/2A/2B), in undifferentiated (d0) and day 5 or 14 differentiated ES cells. Details as in (A). These data show that the TDR deletion does not affect *Airn* splicing suppression but leads to a shortening at the 3′ end. (C) qPCR of unspliced *Airn* in 12.5–13.5 dpc mouse embryos confirms the significant loss of *Airn* steady-state levels at the 3′ end as seen in differentiated ES cells (A,B). Embryos from 3 litters were assayed carrying wildtype (*+/+*, *Thp/+*) or *TDRΔ* (*+*/*TDRΔ*, *Thp/TDRΔ*) paternal alleles. The *Thp* allele carries a deletion of the entire *Igf2r* cluster thus only the paternal allele is present. Samples of the same genotype were averaged and the horizontal lines and error bars show mean and standard deviation. Values for individual embryos are plotted as single data points. The number of samples is given below the genotype (n). Relative *Airn* levels were set to 100% for *+/+*, all others are displayed relative to it. Samples were compared to *+/+* using an unpaired t-test. Details as (A).

While 95% of *Airn* transcripts are unspliced, spliced transcripts comprise 23–44% of the steady-state population due to their increased stability [21]. To confirm the shortening of *Airn* on unspliced and spliced transcripts, we quantified steady-state levels of spliced and unspliced *Airn* in undifferentiated and differentiated *TDRΔ* and control ES cells using qPCR assays spread throughout the *Airn* transcript (Figure 2 map). These qPCR assays allowed us to specifically test if splicing suppression of *Airn* is affected by the TDR deletion. As previously reported, neither spliced nor unspliced *Airn* is expressed in undifferentiated (d0) wildtype (*S12/+*) ES cells but *Airn* is strongly upregulated during differentiation (d5–d14) [31] (Figure 3A, 3B). Similar kinetic behaviour with some biological variation was found for all four *S12/TDRΔ* ES cells using four qPCR assays spaced over the first 73 kb of *Airn*. These were an assay within the first exon of *Airn* that detects both unspliced and spliced *Airn* (START, Figure 3A black bars), two assays that detect only unspliced *Airn* (RP11 at 154 bp and *Airn*-middle at 53 kb, Figure 3A light and dark grey bars) and, two assays that only detect spliced *Airn* (RP6 detecting the SV1a splice variant at 38 kb and RP21 detecting the SV1 variant at 73 kb that are the most abundant spliced products, Figure 3B black and light grey bars). At d14, RP5 detecting the SV2 splice variant at 88 kb showed a significant reduction in 2 of 4 *TDRΔ* clones compared to wildtype (Figure 3B dark grey bars). Two qPCR assays in the 3′ part of *Airn* showed a significant reduction in all four *S12/TDRΔ* cells compared to wildtype cells. These were one assay that detects unspliced *Airn* (*Airn*-end at 99 kb, Figure 3A white bars) and one assay that detects spliced *Airn* (RP4 detecting the SV3 splice variant at 118 kb; Figure 3B white bars). Although splice variants that used an exon 2 located after 73 kb were reduced, the TDR deletion did not induce a major shift in spliced versus unspliced *Airn* transcripts for SV1a and SV1 that end before 73 kb. Thus, the inefficient splicing of *Airn* is not dependent on sequences in the TDRs. In addition, neither the absence of *Airn* transcription in undifferentiated ES cells, nor its ability to be upregulated during differentiation depends on TDR sequences.

We also analysed steady-state levels of *Airn* with or without the TDRs in 12.5–13.5 dpc mouse embryos. We used *+/+* and *+/TDRΔ* embryos and as additional controls, *Thp/+* and *Thp/TDRΔ* embryos. *Thp* is a hemizygous deletion of the *Igf2r* cluster allowing the specific analysis of one parental allele [35]. We analysed unspliced *Airn* with three different qPCR assays at the beginning, middle and end of *Airn* and found that the mouse data largely recapitulate those from the ES cell imprinting model by showing a progressive length reduction towards the 3′ end of *Airn* (Figure 3C). The 5′ assay (RP11 at 154 bp, grey triangles) detected similar amounts of *Airn* from the wildtype and the *TDRΔ* allele. In contrast to the results obtained from the ES cell imprinting model, one assay in the middle of *Airn* (*Airn*-middle at 53 kb, black rectangles) showed a significant reduction to approximately 55% of wildtype levels of *Airn* from the *TDRΔ* allele. The end assay, in agreement with the ES cell imprinting model (*Airn*-end at 99 kb, black circles), detected significant reduction to approximately 5% from the *TDRΔ* allele compared to the wildtype allele. A similar progressive loss of *Airn* towards the 3′ end was detected in the extra-embryonic visceral yolk sac (VYS) (Figure S3A). Together, the quantitative analysis of *Airn* expression supports the conclusion drawn from the genome tiling array, that sequences in the TDR deletion are necessary for full-length *Airn*.

### TDR loss has a minor effect on *Igf2r* imprinted expression

**Figure 4:**
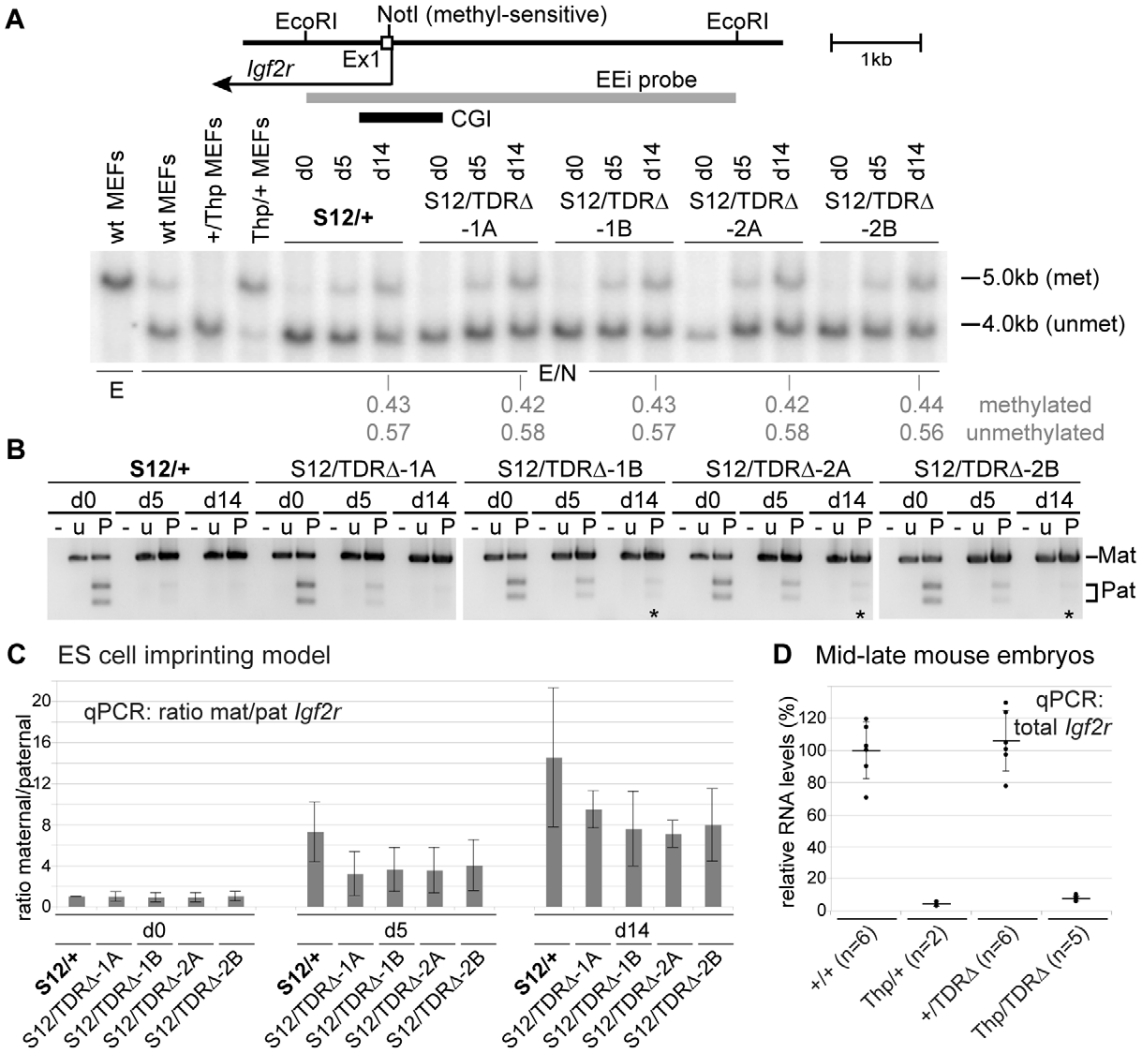
TDR absence has a minor effect on paternal *Igf2r* repression. (A) Genomic DNA digested with EcoRI (E) or EcoRI+methyl-sensitive NotI (E/N) hybridised with probe EEi. wt:wildtype, met:methylated, unmet:unmethylated, *Thp*:deletes the entire *Igf2r* cluster. Quantification of the methylated/unmethylated hybridisation signal shown below for d14, shows equal gain of DNA methylation on the paternal *Igf2r* promoter in *S12/+* and *S12/TDRΔ* cells. Figure S4A shows two further differentiation sets. (B) RT-PCR followed by digestion of a paternal-specific PstI site to assay allelic *Igf2r* expression in ES cells carrying a paternal wild type (*S12/+*) or mutated (*S12/TDRΔ*) allele in four targeted clones. Two further differentiation sets are shown in Figure S4B. -: minus RT, u: undigested, P: PstI digested, Mat: maternal, Pat: paternal. Impaired paternal *Igf2r* repression indicated by the clear presence of two paternal bands at d5 and faint presence at d14 (*) was seen in all four *S12/TDRΔ* cell lines. (C) Allele-specific qPCR quantifying *Igf2r* expression using the same SNP as in (B). The mean maternal∶paternal *Igf2r* expression ratio and standard deviation of three differentiation sets is displayed. As undifferentiated ES cells show biallelic *Igf2r* expression the ratio was set to 1 in *S12/+* d0 cells. Also the *S12/TDRΔ* cells show biallelic expression in undifferentiated ES cells, as the ratio mat/pat is around 1. During differentiation, the ratio in *S12/+* cells increases twofold more compared to *S12/TDRΔ* cells, indicating a compromised although not statistically significantly impaired imprinted expression of *Igf2r* in *S12/TDRΔ* cells. *S12/+* and *S12/TDRΔ* were compared using an unpaired t-test. (D) qPCR of total *Igf2r* steady-state levels in 12.5–13.5 dpc mouse embryos shows a minor loss of paternal *Igf2r* repression. Embryos from 3 litters carrying wildtype (*+/+*, *Thp/+*) or *TDRΔ* (*+/TDRΔ*, *Thp/TDRΔ*) paternal alleles were assayed and compared using an unpaired t-test. The *Thp* allele carries a deletion of the entire *Igf2r* cluster thus only the paternal allele is present. Details as Figure 3C.

In addition to the unusual biology that results in a very long unspliced RNA, the other key property of the *Airn* ncRNA is its *cis*-silencing ability. We have previously shown that shortening *Airn* from 118 kb to 3 kb by targeted insertion of a polyA-signal, leads to a loss of repression of the overlapped *Igf2r* gene and the upstream flanking *Slc22a2* and *Slc22a3* genes [19]. As TDR deletion led to a 3′ shortening of *Airn* we first asked if this affected its ability to repress *Igf2r*. This can be monitored indirectly by the gain of DNA methylation on the paternal *Igf2r* promoter-associated CGI or, directly by assaying allelic *Igf2r* expression. We first analysed gain of DNA methylation by methyl-sensitive restriction digestion of genomic DNA from undifferentiated and differentiated wildtype (*S12/+*) and *TDRΔ* (*S12/TDRΔ*) ES cells (Figure 4A). In undifferentiated wildtype ES cells both *Igf2r* parental alleles are methylation-free as demonstrated by the single 4 kb band. Upon differentiation a 5 kb band indicating a gain of DNA methylation on the paternal allele appears, which increases in strength during differentiation. At d14, quantification from three independent differentiation sets (Figure 4A, Figure S4A) revealed methylated∶unmethylated ratios of 0.75∶1 in wildtype ES cells and a similar ratio in four *TDRΔ* ES cells. This indicates the gain of DNA methylation on the repressed paternal *Igf2r* promoter is not dependent on the TDRs.

We next directly analysed *Igf2r* imprinted expression using the SNP that lies in *Igf2r* exon 12, 20 kb upstream to the ICE, which can be distinguished by PstI digestion (Figure 4B, Figure S4B). In undifferentiated (d0) wildtype ES cells, PstI digested cDNA results in an undigested maternal (Mat) fragment and two restriction fragments representing the paternal (Pat) allele, indicating biallelic *Igf2r* expression. As previously described, the paternal-specific fragments are gradually lost during differentiation, which indicates maternally-biased *Igf2r* imprinted expression [31]. Undifferentiated ES cells with a paternal *TDRΔ* allele (*S12/TDRΔ*) also express *Igf2r* biallelically. However, they differ from wildtype cells by showing reduced paternal *Igf2r* repression during differentiation, as the two paternal-specific restriction fragments are more visible at d5 and in some cases, remain visible at d14. We quantified this effect on paternal *Igf2r* repression using a qPCR assay that uses forward primers specific for the two SNP alleles in combination with a common reverse primer. The ratio of the maternal to the paternal allele in undifferentiated wildtype ES cells was set to 1 (Figure 4C), as they were shown previously to express *Igf2r* biallelically [31]. Wildtype ES cells show a consistent increase in the maternal to paternal *Igf2r* ratio during differentiation, representing specific upregulation of the maternal allele, with constant low-level paternal expression. Undifferentiated ES cells with a paternal *TDRΔ* allele (*S12/TDRΔ*) also show ratios close to 1, indicating biallelic *Igf2r* expression. *S12/TDRΔ* cells show an increased maternal∶paternal *Igf2r* ratio during differentiation, however, this only reached 44–55% at d5 and 49–65% at d14 of the ratio seen in wildtype cells, representing an approximate 2-fold upregulation of the paternal *Igf2r* allele in *TDRΔ* cells compared to wildtype (Figure 4C). However, neither the maternal∶paternal *Igf2r* ratio, nor total *Igf2r* levels (data not shown) were statistically different in *TDRΔ* cells compared to wildtype cells. Total *Igf2r* levels were then analyzed in 12.5–13.5 dpc mouse embryos carrying a paternal *TDRΔ* (*+/TDRΔ*, *Thp/TDRΔ*) or paternal wildtype (*+/+*, *Thp/+*) chromosome (Figure 4D). Mean total *Igf2r* levels in +/+ embryos were set to 100% and *+/TDRΔ* embryos showed an average of 106%. As the majority of *Igf2r* transcripts are produced from the maternal wildtype allele that could mask changes on the paternal allele, we analysed embryos carrying a maternal *Thp* deletion allele that only have the paternal *Igf2r* allele. The wildtype chromosome in *Thp/+* embryos showed 4.5% of levels in +/+ embryos while the *TDRΔ* chromosome in *Thp/TDRΔ* embryos showed 7.6% of wild type levels, representing a 1.7-fold upregulation of *Igf2r* from the paternal *TDRΔ* allele that was however, not statistically significant. In extra-embryonic tissues, in addition to *Igf2r*, the *Slc22a2* and *Slc22a3* genes show *Airn*-dependent imprinted expression [19]. Analysis of all three genes in VYS shows a similar trend for a modest but not consistently significant loss, of paternal repression upon paternal transmission of the *TDRΔ* allele (Figure S3B–S3D). Together this indicates a similar trend for a minor loss of imprinted repression of protein-coding genes in both ES cells and mid-late gestation embryos and extra-embryonic tissues, indicating that deletion of the TDRs slightly reduces the repressor efficiency of *Airn*.

### TDRs are required for the regulation of ICE DNA methylation

**Figure 5:**
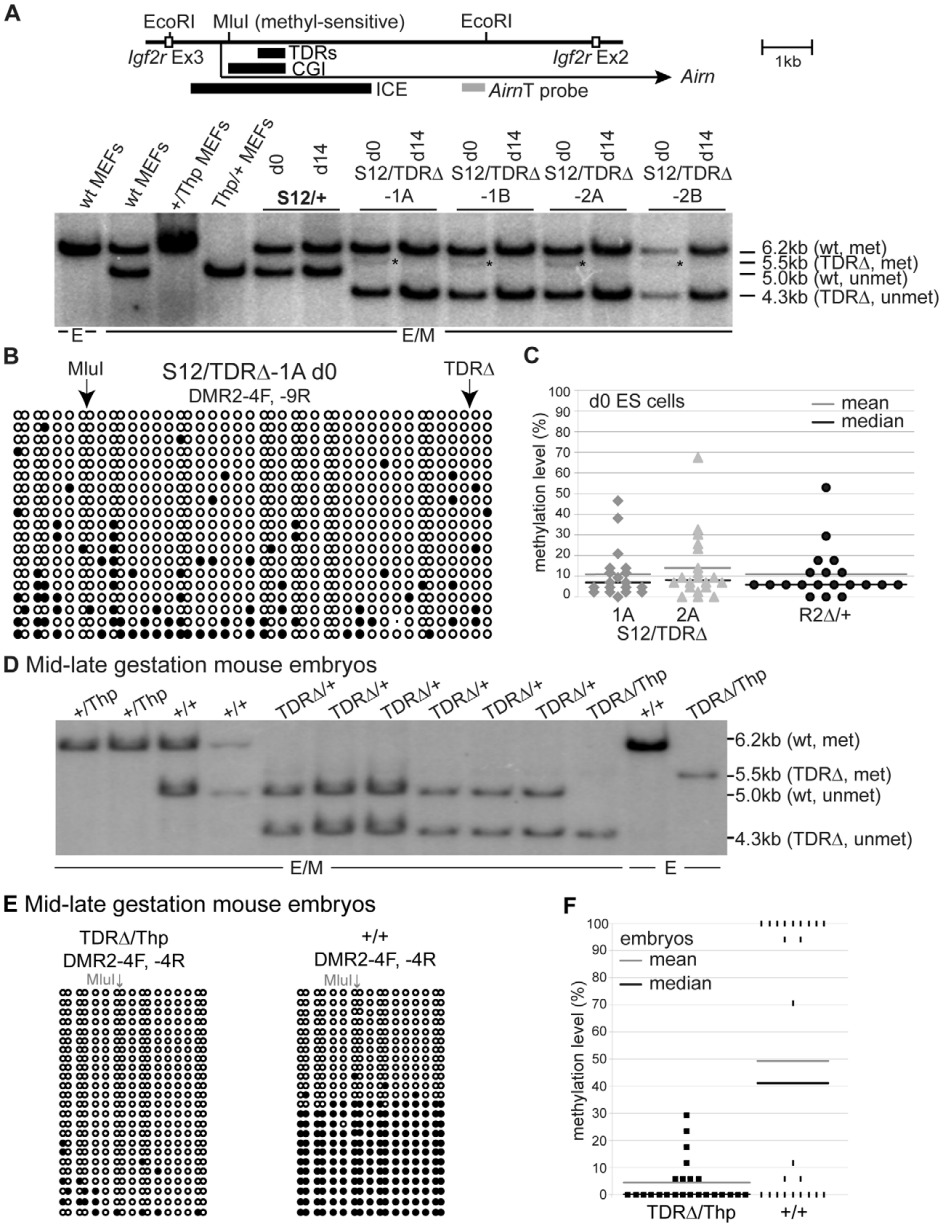
TDRs are required for the regulation of ICE DNA methylation. (A) Genomic DNA digested with EcoRI (E) or EcoRI+methyl-sensitive MluI (E/M) and hybridised with probe *Airn*T. Cells carrying a paternal *TDRΔ* (*S12/TDRΔ*) or wildtype (*S12/+*) allele have a 6.2 kb band from the maternally-methylated plus 4.3 kb or 5.0 kb from the paternally-unmethylated allele. *S12/TDRΔ* cells additionally show at d0, a faint 5.5 kb band representing a paternal partially methylated allele (*) that is lost by d14 in differentiated cells. (B) Bisulfite sequencing of undifferentiated *S12/TDRΔ*-1A ES cells. Each row represents one allele, single columns: one CpG, double columns: two neighbouring CpGs. White/black circles: unmethylated/methylated CpGs. Arrows: position of MluI site and *TDRΔ* deletion. Primers DMR2-4F and -9R specifically amplify the paternal allele in *S12/TDRΔ* ES cells (details in Figure S5B). Bisulfite sequencing confirms a low level of DNA methylation on the paternal *TDRΔ* allele at d0 only. (C) Percent methylation levels for two *S12/TDRΔ* and control *R2Δ/+* ES cells. The *R2*Δ deletion removes the maternal ICE, thus only the wildtype paternal ICE is detected. Each point represents one sequenced clone and shows that low-level DNA methylation is a general feature of the paternal ICE in d0 ES cells and not a consequence of the TDR deletion. (D) DNA blot analysing the MluI methylation status as in (A) for 12.5–13.5 dpc embryos carrying a maternal *TDRΔ* (*TDRΔ/+, TDRΔ/Thp*) or wildtype (*+/+, +/Thp*) allele. The 5.0 kb methylated band is faint or absent in *TDRΔ/+* embryos, but present in *+/+* embryos (6.2 kb), showing that maternal inheritance of the *TDRΔ* allele leads to loss of DNA methylation. (E) Bisulfite sequencing of embryonic genomic DNA (details as in B) shows that maternal transmission of the TDR deletion leads to a major loss in DNA methylation. For *TDRΔ/Thp* only the maternal allele, for *+/+* both parental alleles were analysed. (F) Percent methylation level for *TDRΔ/Thp* and *+/+* embryos as in (C).

The *Airn* CGI is contained within the ICE that carries a gametic DNA methylation imprint on the maternal allele while the paternal allele is free of methylation. This gametic methylation imprint is present in undifferentiated ES cells as they are derived from the inner cell mass of the 3.5 dpc blastocyst [31]. To test if TDR deletion from the paternal allele compromised the methylation-free state of the paternal ICE, we analysed genomic DNA from undifferentiated (d0) and differentiated (d5 and/or d14) *S12/+* and *S12/TDRΔ* ES cells by methyl-sensitive restriction digestion of genomic DNA (Figure 5A). Wildtype (*S12/+*) undifferentiated and differentiated ES cells both show a 6.2 kb band originating from the methylated maternal allele and a 5.0 kb band from the unmethylated paternal allele. In *S12/TDRΔ* ES cells, two fragments were similarly present, the 6.2 kb fragment from the wildtype maternal allele and a 4.3 kb fragment from the unmethylated paternal allele that is shortened by the TDR deletion. In addition, a faint but reproducible 5.5 kb band that must originate from a methylated *TDRΔ* paternal allele (Figure S1) was detected in undifferentiated but not in differentiated *S12/TDRΔ* ES cells (*Figure 5A). This indicates a transient gain of DNA methylation in undifferentiated *S12/TDRΔ* ES cells that is lost during differentiation. Figure S5A shows two additional differentiation sets with similar behaviour. To test if low-level DNA methylation on the paternal ICE represents a property of undifferentiated ES cells, rather than a consequence of the TDR deletion, we performed bisulfite sequencing, specifically analysing the paternal allele, in undifferentiated *S12/TDRΔ*-1A and -2A ES cells and a control ES cell line with the ICE deleted from the maternal allele (*R2Δ/+*) [36]. Figure 5B, 5C and Figure S5C–S5E show that low-level DNA methylation from 11–14% with extremes ranging from 0–64%, is a general feature of the paternal ICE in undifferentiated ES cells and not a consequence of the TDR deletion. This low-level DNA methylation is however transient as the ICE becomes methylation-free in differentiated ES cells and in differentiated primary embryonic cells (Figure 5A, Figure S5A, S5D, S5E).

A possible effect of the TDR deletion on the methylated state of the maternal allele cannot be analysed in the ES cell imprinting model, as homologous recombination with the targeting vector replaces the CpG methylated genomic DNA with unmethylated DNA grown in bacteria. We therefore used *TDRΔ* embryos to analyse DNA methylation of the ICE by methyl-sensitive restriction digestion of genomic DNA. Paternal transmission of the *TDRΔ* did not affect the maintenance of the unmethylated state, confirming the data obtained from the ES cell imprinting model (data not shown). In contrast, maternal transmission of the *TDRΔ* led to almost complete loss of the methylated 5.5 kb fragment (detected as 6.2 kb in wildtype mice, Figure 5D). Bisulfite sequencing of mouse embryos carrying a maternal *TDRΔ* (*TDRΔ*/+ and *TDRΔ/Thp*) or a wildtype maternal allele (*+/+*) confirms that maternal transmission of the *TDRΔ* allele led to near complete loss of the maternal methylation imprint (Figure 5E, 5F, Figure S5F). The *TDRΔ* allele showed mean methylation levels of only 4% with extremes ranging from 0–29%. Together these results demonstrate that the TDRs do not play a role in the maintenance of the methylation-free state of the paternal ICE but are essential for methylation on the maternal ICE. Whether the TDRs play a role in the acquisition of the maternal ICE methylation mark in oocytes or its maintenance at later developmental stages was not determined. The loss of maternal ICE methylation in *TDRΔ*/+ embryos and VYS, resulted in maternal expression of the same progressively shorter *Airn* transcript with similar ability to repress *Igf2r*, *Slc22a2* and *Slc22a3* (Figure S3E–S3J), as shown above for a paternal *TDRΔ* allele. Neither paternal nor maternal TDR inheritance has an effect on viability or fertility, examining respectively 29 and 65 offspring. In addition TDR homozygotes are obtained in the expected ratio from double heterozygote crosses (*i.e.*, 19 wildtype, 34 heterozygotes and 12 homozygotes were found in 65 offspring). However, although male TDR homozygotes are fertile and produce viable young (16 offspring from 4 litters), female TDR homozygotes show reduced fertility and do not produce viable offspring (10 offspring were obtained from 4 litters but all died within 2 days, indicating a role for *Igf2r* in the female reproductive tract as noted earlier [37]. We have previously shown that low levels of *Igf2r* that continue to be expressed from the paternal allele in wildtype embryos (approximately 5%, see *Thp*/+ in Figure 4D) are not sufficient for viability in the absence of a maternal *Igf2r* allele [37]. Live born fertile *TDRΔ* offspring are obtained with *Igf2r* levels that average 16% (ranging from 11–21%) of wildtype at 12.5–13.5 dpc (see Figure S3F). These crosses contain 129Sv and C57BL/6J genotypes and additional contribution to the survival of *TDRΔ*/+ mice could come from a mixed genetic background, which was previously shown to influence viability upon loss of maternal *Igf2r* contribution [37], [38].

### Generation of CGI deletion ES cells

The above data shows that the TDRs that lie in the 3′ half of the CGI (Figure 1B), act on the paternal chromosome to control the full-length of *Airn* and on the maternal chromosome to regulate ICE DNA methylation. To determine if the CGI contains additional elements regulating *Airn* expression we next removed the entire CGI. The same wildtype parental (*S12/+*) ES cells were used to delete a 1129 bp fragment, starting 177 bp downstream of the *Airn*-TSS and ending at the same position as for the TDR deletion (Figure S6A). This deletion left behind 106 bp of the 5′ part of the CGI including the diagnostic MluI site, however the remnant is too small to fit conventional CGI definition criteria [39]. The selection cassette was inserted at the same position as for the TDR deletion to avoid leaving a loxP site at the deletion site. We obtained two homologously-targeted clones that were both targeted on the paternal allele (*S12/CGIΔ+cas*-1, -2) (Figure S6B, S6C). The selection cassette was removed by transient CRE expression and four ES cell subclones (*S12/CGIΔ*-1A/-1B/-2A/-2B) were used for analysis in the ES cell imprinting model (Figure 1A, Figure S6B).

### The CGI plays a major role in *Airn* length

**Figure 6:**
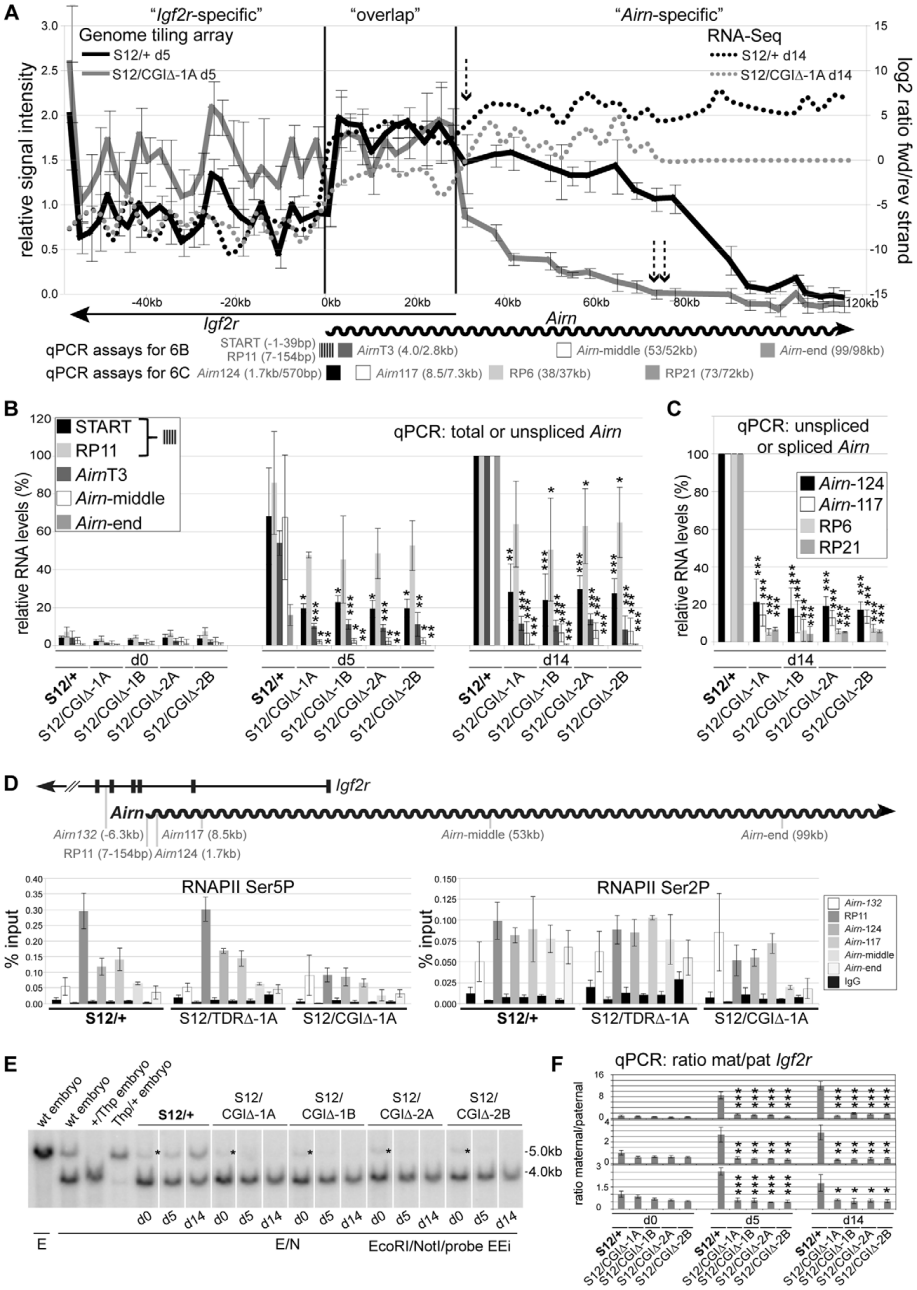
The *Airn* CGI plays a major role in *Airn* transcription and function. (A) *Airn* expression by genome-tiling array (left axis) and strand-specific expression analysis by RNA-Seq (right axis) for differentiated *S12/+* and *S12/CGIΔ*-1A cells. Dashed arrows: sharp drop of *Airn* hybridisation signals in the *Airn*-specific region (single) and absence after 73 kb (doublet). Below: qPCR assays relative to *Airn*-TSS with colour code as (B,C). Striped box: overlapping START+RP11 assays. (B) qPCR of total+unspliced *Airn* in d0/d5/d14 differentiated *S12/+* and four *S12/CGIΔ* clones shows unspliced *Airn* is reduced by ∼40% at the 5′ end (RP11/154 bp), but when assayed downstream (*Airn*-middle/53 kb, *Airn*-end/99 kb) or at positions which include splice variants (START), is reduced by >70% in *S12/CGIΔ* cells. Shown are mean and standard deviation of three differentiation sets (details as Figure 3A). (C) *Airn* qPCR in *S12/+* and four *S12/CGIΔ* d14 clones shows that unspliced *Airn* is reduced by 79–83% at 0.57 kb and ∼85% at 7.3 kb, while spliced *Airn* reduced by >85%. Shown are mean and standard deviation of three differentiation sets (details as Figure 3A). (D) ChIP for Ser5P/Ser2P RNAPII in *S12/+*, *S12/TDRΔ*-1A and *S12/CGIΔ*-1A d11 cells shows unaffected *Airn* initiation and elongation (except at *Airn*-end) in *TDRΔ* and a sharp RNAPII decrease in the *CGIΔ* allele. The mean and standard deviation of three technical replicates is shown. Assay *Airn*-132 controls for background from the overlapping *Igf2r* transcript, which is 2-fold higher in *CGIΔ* that fails to repress the paternal *Igf2r* promoter. Map for qPCR assays as Figure 2. (E) DNA blot analysing methylation of the *Igf2r* promoter NotI site (see Figure 4A). *methylated fragment in d0 cells originating from feeder-cells. This blot shows that cells carrying a paternal *CGIΔ* allele contrary to wildtype cells do not gain the methylated 5 kb band on the paternal *Igf2r* promoter. White lines: indicate the order of samples run on the same gel was changed electronically. (F) qPCR quantifying allelic expression shows absence of *Igf2r* imprinted expression (Mat∶Pat ratio is close to 1), in four *CGIΔ* (*S12/CGIΔ*) cell lines compared to wildtype (*S12/+*). Three differentiation sets are shown separately due to variability in Mat∶Pat ratios in wildtype controls for each set. Bars represent the mean, error bars the standard deviation of 3 technical replicates (details as Figure 4C).

To test if the CGI deletion enhanced the shortening of the *Airn* transcript observed after the TDR deletion, we differentiated *S12/+* and *S12/CGIΔ*-1A ES cells and analysed them by RNA hybridization to genome tiling arrays (Figure 6A). In contrast to the *S12/+* cells (and *S12/TDRΔ* cells in Figure 2), the relative signal intensities from *S12/CGIΔ* cells did not increase at the transition from the ‘*Igf2r*-specific’ to the ‘overlap’ region but instead were similar. Since signals in the overlap region are derived from both *Igf2r* and *Airn*, this indicated an absence of *Airn* transcription in this region or a shortening of *Airn* not resolved on the array. In addition, *S12/CGIΔ* cells showed a sharp drop in signal intensity at the start of the ‘*Airn*-specific’ region and signals were not detected after 73 kb downstream of the *Airn* transcription start (respectively single and double dashed arrows, Figure 6A). This was in contrast to *Airn* in *TDRΔ* cells that showed a drop in signal intensity from 68 kb onwards and no signal only after 90 kb (Figure 2). Lastly, higher signal intensities in the *Igf2r*-specific region in *S12/CGIΔ* cells compared to wildtype, indicate a gain of bi-allelic *Igf2r* expression. We also performed strand-specific RNA-Seq and plotted the log2 ratio of the number of reads originating from the forward and reverse strand to obtain an estimate for strand-specific expression in the analysed region (Figure 6A, dotted lines). In *S12/+* cells, reads in the ‘*Igf2r*-specific’ region show specific expression of *Igf2r*. In the ‘overlap’ region, the ratio then shifts towards the *Airn*-expressing forward strand, which is even more pronounced in the ‘*Airn*-specific’ region. In *S12/CGIΔ* cells, a similar albeit less pronounced shift in the ratio is seen upon transition from the ‘*Igf2r*-specific’ to the ‘overlap’ region, with a further shift occurring at the transition from the ‘overlap’ to the ‘*Airn*-specific’ region that is not detected after 73 kb and is reduced compared to *S12/+* cells. This confirms in a strand-specific manner, a low level but persistent *Airn*-expression upon deletion of the CGI. Together, these data indicate that high *Airn* expression and production of full-length *Airn* transcripts and as a consequence, the ability to repress *Igf2r in cis*, are dependent on the CGI.

To validate the genome tiling array data we analysed cDNA from undifferentiated and differentiated wildtype (*S12/+*) and *CGIΔ* (*S12/CGIΔ*) ES cells using five qPCR assays spaced along the length of *Airn*. In undifferentiated ES cells with and without the CGI, *Airn* expression was mostly absent consistent with the previously observed lack of *Airn* expression in undifferentiated ES cells [31]. The low level of *Airn* expression seen in undifferentiated ES cells in Figure 6B represents a small amount of spontaneous differentiation that was similar in wildtype and *CGIΔ* ES cells. During differentiation *Airn* was upregulated in wildtype ES cells using all five qPCR assays. In contrast, the *CGIΔ* ES cells showed consistently less *Airn* at all analysed positions (Figure 6B). However, the extent of the loss of steady-state levels differed along the length of the transcript. The START assay showed that total unspliced and spliced *Airn* is reduced to an average of 24–30%. Unspliced *Airn* detected with RP11 at 154 bp downstream of the TSS showed an average reduction to 50–65%, which was statistically significant in 3 of 4 clones at d14. This difference between total and unspliced *Airn* is explained by the major loss of the *Airn* splice variants that require transcription elongation to at least 72 kb (Figure 2), and represent up to 44% of steady-state *Airn* levels [21]. Unspliced *Airn* detected with the *Airn*T3 assay at 2.8 kb from the TSS on the *CGIΔ* allele showed an average reduction to 8–14% and detection by the *Airn*-middle assay at 52 kb from the TSS showed reduction to 6–8% (note that distances from the TSS on the *CGIΔ* allele are reduced by 1129 bp). Finally at *Airn*-end (98 kb from the *Airn*-TSS) the reduction was to 0–0.4% of wildtype levels. This indicates that the CGI deletion induced successive loss of *Airn* with increasing distance from the 5′ end.

To further map the observed shortening of *Airn* we used two more assays at the 5′ end (Figure 6C). In *CGIΔ* cells, the *Airn*-124 assay at 570 bp from the TSS showed an average reduction of *Airn* steady-state levels to 17–21%, while the *Airn*-117 assay at 7.3 kb from the TSS showed an average reduction to 13–14%. We also analysed steady-state levels of two *Airn* splice variants to see if the splicing suppression was altered by the CGI deletion (Figure 6C). The RP6 assay showed the SV1a splice variant is reduced on average to 5–7% of wildtype levels, while the RP21 assay showed the SV1 splice variant is reduced on average to 4–7%. Both these splice variants require transcription elongation to 72 kb (Figure 2). Splicing suppression was therefore not altered after the CGI deletion as the abundance of splice variants decreased in a similar manner as unspliced *Airn*. Furthermore, the abundance of splice variants from the *CGIΔ* allele is at most 7% of wildtype levels. As splice variants represent up to 44% of the *Airn* steady-state population, this indicates that the 24–30% of steady-state levels observed with the START assay (Figure 2B) represent more than 60% of initiating transcripts, confirming the result obtained for the RP11 assay. Together the data shows that *Airn* full-length elongation is significantly affected by deletion of the CGI with a successive loss of *Airn* with increasing distance from the 5′ end. However, by analysing RNA steady-state levels by qPCR a more moderate change is seen in transcription initiation such that 50–65% of *Airn* transcripts elongate at least to 154 bp.

To test if the observed decrease of *Airn* ncRNA expression was reflected by altered recruitment of RNA polymerase II (RNAPII) we performed chromatin immunoprecipitation using antibodies specifically recognising initiating RNAPII phosphorylated at the Serine 5 (Ser5P) residue of its carboxy-terminal domain (CTD) and elongating RNAPII phosphorylated at Serine 2 (Ser2P) of its CTD. RNAPII occupancy in differentiated *S12/+*, *S12/TDRΔ-1A* and *S12/CGIΔ-1A* ES cells was analysed at five positions along the gene body of *Airn* as well as in intron 5 of *Igf2r* to control for overlapping *Igf2r* transcription (Figure 6D map). Whereas equal amounts of RNAPII Ser5P were found in *S12/+* and *S12/TDRΔ-1A* cells, it was strongly reduced at the *Airn* 5′ region in *S12/CGIΔ-1A* cells, indicating reduced *Airn* transcriptional initiation on the *CGIΔ* allele (Figure 6D left). For RNAPII Ser2P, *S12/+* and *S12/TDRΔ-1A* showed similar enrichment except for *Airn*-end, where *S12/TDRΔ-1A* showed reduced levels. In *S12/CGIΔ-1A* cells, RNAPII Ser2P levels were increased in intron5 of *Igf2r* consistent with the increase in *Igf2r* levels observed in Figure 6A. RNAPII Ser2P levels within the *Igf2r/Airn* transcriptional overlap were lower compared to the other two cell lines indicating that *Airn* transcriptional elongation on the *CGIΔ* allele is strongly reduced (Figure 6D right). An independent RNAPII ChIP experiment showed a similar result (data not shown). Together, the analysis of RNA levels and RNAPII occupancy indicate that the CGI which is localised downstream of the *Airn* promoter, controls *Airn* initiation and elongation.

### *Airn* CGI deletion results in biallelic expression of *Igf2r*

As the majority of *Airn* transcripts were only between 154–570 bp long (Figure 6C) we tested if *Airn* produced from the *CGIΔ* allele was unable to silence *Igf2r*, as expected from previous experiments that truncated *Airn* to 3 kb from the TSS [19]. We first analysed the DNA methylation status on the paternal *Igf2r* promoter-associated CGI, as described in Figure 4A for the *TDRΔ*. Figure 6E and Figure S7 show that in contrast to wildtype ES cells, all four *CGIΔ* ES cell lines fail to gain DNA methylation on the paternal *Igf2r* promoter during differentiation, indicative of biallelic *Igf2r* expression in these cells. Next, we performed allelic expression analysis of *Igf2r* using the qPCR assay described above for the TDR deletion in Figure 4C. The results (Figure 6F) show that all differentiated *CGIΔ* cells displayed an unchanging maternal∶paternal expression ratio during differentiation indicative of biallelic *Igf2r* expression. This contrasts to wildtype cells that show an increasing ratio of maternal∶paternal *Igf2r* expression during differentiation indicative of maternally-biased imprinted expression. An absence of *Igf2r* imprinted expression can also be inferred from the tiling array analysis in Figure 6A where increased *Igf2r* hybridization signals are seen in the *Igf2r*-specific region and from the increased RNAPII occupancy in *Igf2r* intron 5 in Figure 6D. Thus, these results show that *Airn* transcripts in *S12/CGIΔ* ES cells, the majority of which had a length of between 154–570 bp are as expected, defective in their ability to silence *Igf2r*.

### Paternal ICE methylation-free state depends on the CGI

**Figure 7:**
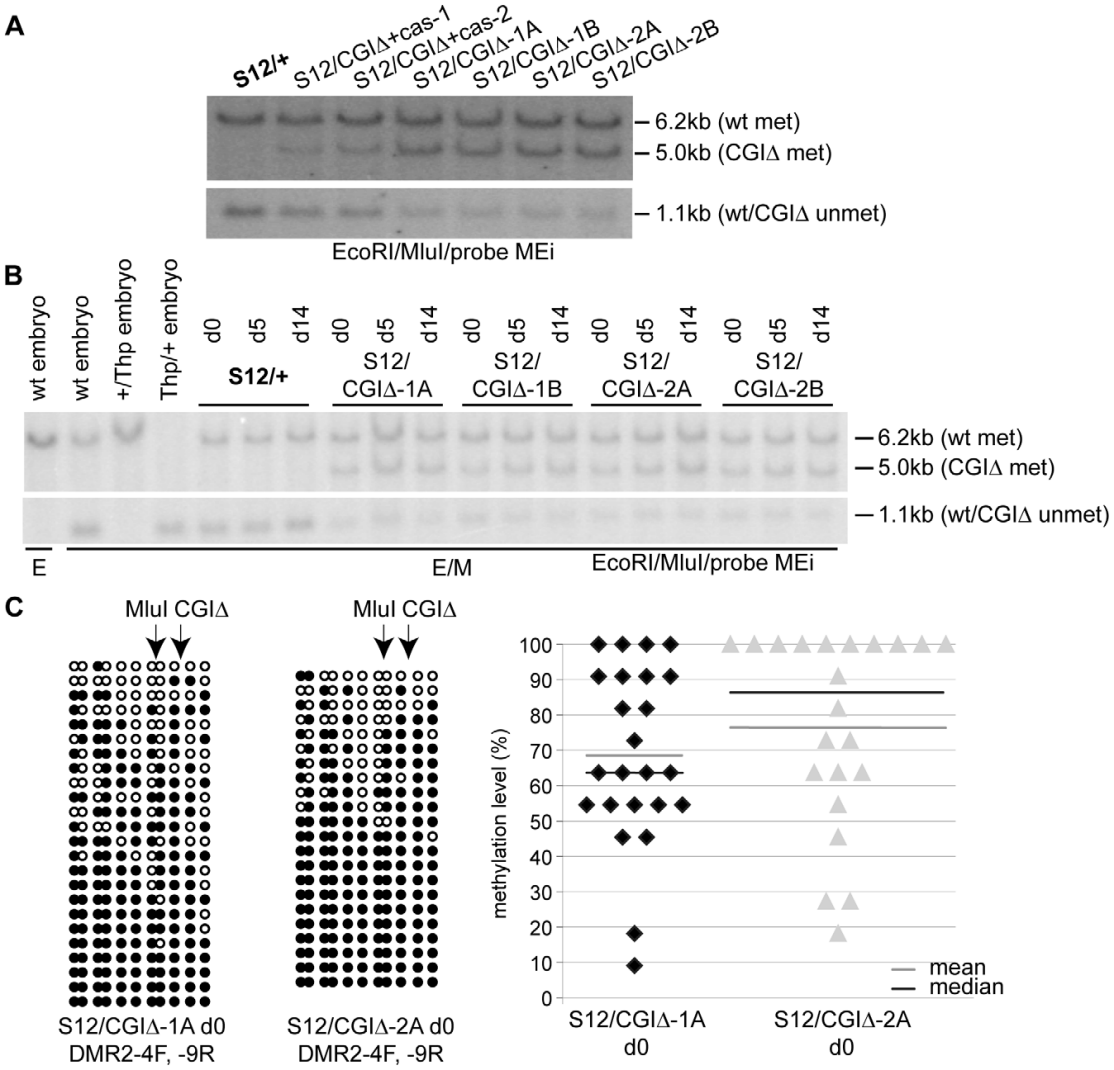
The methylation-free state of the paternal ICE depends on the CGI. (A) DNA blot assaying methylation of the *Airn* promoter MluI site as in Figure 5A, in undifferentiated ES cells carrying a paternal *CGIΔ* or wildtype (*+*) allele. The 5.0 kb band identified by probe MEi (see Figure 5A map) indicates a gain of methylation on the *CGIΔ* paternal allele. This band is weaker in cells with lower passage numbers that still retain the selection cassette (*S12/CGIΔ+cas*-1,-2) compared to cells that have been in culture for 8 more passages (*S12/CGIΔ*-1A,-1B,-2A,-2B) with a deleted selection cassette. The lower panel confirms this by showing a matching loss of the unmethylated 1.1 kb fragment specific to the paternal allele in cells with a higher passage number. Both panels were from the same blot and the intervening area lacking any hybridisation signal removed. (B) DNA blot as in (A) assaying *Airn* promoter MluI methylation during ES cell differentiation showing that the level of paternal methylation on the *CGIΔ* allele in undifferentiated ES (d0) cells (5 kb band) does not change in differentiated d5 and d14 cells. Probe MEi is a 1 kb EcoRI-MluI fragment shown in Figure 5A map. (C) Bisulfite sequencing of two undifferentiated *S12/CGIΔ* ES cell clones using primers spanning the deletion that specifically amplify the paternal *CGIΔ* allele, confirms the strong gain of DNA methylation, but also shows that some alleles are more methylated than others (details as Figure 5B).

Finally, we tested if deletion of the CGI affected the methylation-free state of the *Airn* promoter region on the normally unmethylated paternal allele. The CGI deletion left behind 106 bp of the 5′ part of the CGI including the diagnostic MluI site analysed for the TDR deletion in Figure 5A. Undifferentiated ES cells with a wildtype paternal allele (*S12/+*) showed a 6.2 kb maternally methylated and an equally strong 1.1 kb paternally unmethylated band (Figure 7A). Cells with a paternal CGI deletion (*S12/CGIΔ*) showed a wildtype 6.2 kb maternally-methylated fragment and a 5 kb paternally-methylated band. The size of the paternal fragment is reduced to 1.1 kb when unmethylated. In Figure 7A we also examined *CGIΔ* cells with (*S12/CGIΔ+cas*) and without (*S12/CGIΔ*) the selection cassette, to obtain information from cells that had experienced a short and long culture period since the loss of the CGI. Compared to *S12/CGIΔ+cas* cells, *S12/CGIΔ* cells that lack the selection cassette have been an additional 8 passages in culture and show an increased intensity of the 5.0 kb band indicating that DNA methylation increases with passage number. However, in contrast to the TDR deletion shown in Figure 5A, DNA methylation was not lost upon differentiation as indicated by the similar intensity of the 5 kb band in d0 and d14 *S12/CGIΔ* cells (Figure 7B, S8). Bisulfite sequencing was used to determine the extent of DNA methylation on the *CGIΔ* allele in undifferentiated ES cells using primers spanning the deletion that specifically amplify the paternal *CGIΔ* allele (Figure S5B). In two *S12/CGIΔ* ES cell lines the results show a high level of DNA methylation (Figure 7C left). The % methylation levels were 69–76%, with extremes ranging from 9–100% (Figure 7C right). Taken together, this analysis shows that deletion of the CGI leads to a strong non-reversible gain of DNA methylation *in cis*, indicating that one major function of the CGI on the paternal allele is to block DNA methylation on the paternal ICE.

## Discussion

This study assessed the possible role played by the *Airn* CpG island (CGI) and a region of tandem direct repeats (TDRs) on the *Airn* transcript and the allelic methylation state of the ICE that controls *Airn* expression. Since these elements lie immediately downstream of the *Airn* transcription start and thus are present on the *Airn* transcript, they may also play a role in *Airn* biology. Our results using two targeted deletions analyzed in an ES cell imprinting model and in knockout mice, show that the CGI regulates both initiation and elongation efficiency of the *Airn* promoter and is also necessary to maintain the unmethylated state of the paternal ICE. This indicates the existence of a new transcriptional role for CGIs in the mammalian genome acting downstream of the promoter and transcription start site. In contrast, the TDRs that occupy the second half of the CGI play a minor role in *Airn* transcriptional processivity but are essential for methylation of the maternal ICE that represses the *Airn* promoter on this chromosome.

### The *Airn* downstream CGI controls its length and thereby its silencing ability

The 1129 bp deletion of the complete *Airn* downstream CGI had a moderate effect on the most 5′ RNA levels such that two qPCR assays within the first 154 bp downstream of the *Airn*-TSS detected approximately 50–65% of wildtype levels of the normally 118 kb long *Airn* ncRNA. *Airn* transcripts were reduced to ∼14% between 1.7–8.5 kb and to ∼6% between 53–73 kb, while no transcripts were detected at 99 kb. In addition, both initiating and elongating forms of RNAPII were reduced compared to control cells. In contrast the TDR deletion had a minor effect on the length of *Airn* with transcripts at normal levels for the first two-thirds, but progressively reduced after 68 kb and absent at 99 kb, with the elongating form of RNAPII reduced at the 3′ end of *Airn*. This indicates that the efficiency of RNAPII to elongate *Airn* over 118 kb is regulated by the CGI and at least in part, also by the TDRs. Notably, in view of the splicing suppression of wildtype *Airn* that results in splicing of only 5% of transcripts [21], the production of all four splice variants was decreased in proportion to the unspliced transcripts indicating that neither the CGI nor TDRs cause splicing suppression.

We have previously shown that *Airn* must be longer than 3 kb and be expressed from a strong promoter, to induce silencing of the overlapped *Igf2r* promoter [19], [32]. A loss of paternal *Igf2r* repression after the CGI deletion that shortened the majority of *Airn* transcripts to less than 0.5% of its normal length is therefore expected, and this deletion was only analysed in the ES cell imprinting model. The TDR deletion although producing normal levels of the *Airn* transcript that overlap the 28 kb distant *Igf2r* promoter and elongate up to 90 kb from the *Airn*-TSS, nevertheless showed a minor loss of paternal *Igf2r* repression. This was seen as a 1.7–2.0 fold upregulation of the paternal *Igf2r* allele in differentiated ES cells and in mid-late gestation embryos and VYS, which was not statistically significantly different from paternal repression on wildtype chromosomes. A similar minor increase in paternal steady-state levels was observed for *Slc22a2* and *Slc22a3* in the VYS. We lack an explanation for this minor effect. It appears not to arise from changed developmental kinetics of *Airn* expression that were similar in wildtype and *TDRΔ* differentiating ES cells, but it may reflect changes in RNAPII post-translational modification not detected with current antibodies. Currently it is unknown if the *Airn* ncRNA or the act of its transcription induce imprinted expression of *Igf2r* [16], [40]. For the *Slc22a3* gene that lies 275 kb downstream of *Igf2r* and is repressed by *Airn* only in placenta, *Airn* was shown to localize to the *Slc22a3* promoter and to induce imprinted expression by interacting with the G9A histone methyltransferase. However, imprinted expression of *Igf2r* was not affected in these studies [41] or in studies eliminating PRC2 activity [42]. The results obtained here do not distinguish between a role for the *Airn* ncRNA or its transcription, but are in agreement with previous analyses that demonstrated a role for high *Airn* expression and a length longer than 3 kb to repress *Igf2r in cis* [21], [32].

The *Xist* macro ncRNA that induces whole chromosome silencing in female XX mammals has been suggested to share similarities with imprinted repressor ncRNAs such as *Airn* and *Kcnq1ot1* [43], [44]. Notably *Xist* contains a set of 5′ direct ‘A’ repeats that are essential for *Xist* to induce chromosome silencing [45]. The *Airn* TDRs may have served a similar purpose. Here we show that the TDRs are not required for *Airn* to repress its target genes as despite the minor loss of paternal repression, imprinted expression is present in *TDRΔ* cells and mice. However, since the TDRs are required for maternal ICE methylation, they are necessary to ensure expression of the maternal *Igf2r* allele as it has been previously shown that mouse embryos lacking maintenance DNA methylation, repress both parental *Igf2r* alleles [22]. The imprinted *Kcnq1ot1* macro ncRNA shares many features with the *Airn* ncRNA and its TSS lies on the 5′ border of the CGI (Figure 1C), which contains a series of TDRs that lack sequence conservation with those in *Airn* [30], [46]. Two overlapping deletions have been used to test the function of this region in the *Kcnq1ot1* ncRNA. The first is a 657 bp deletion starting just downstream of the *Kcnq1ot1*-TSS [18], while the second deletion removed 890 bp and overlapped 40% of the 657 bp deletion [47]. The ability of the deleted *Kcnq1ot1* ncRNA to repress flanking genes on the paternal chromosome was found to be unchanged in midgestation embryos for the 657 bp deletion. However, the 890 bp deleted *Kcnq1ot1* allele showed a failure to repress some genes in this cluster in a lineage-specific manner that correlated with failure to gain DNA methylation on the derepressed genes. Although the failure to repress flanking genes was attributed to a failure in recruiting DNMT1 due to the lack of the 890 bp region in the ncRNA [47], both *Kcnq1ot1* and *Airn* are able to repress genes in mouse embryos lacking the *Dnmt1* gene that are deficient in genomic methylation [29], [48]. The TDR deletion described here resulted in loss of the 3′ part of *Airn* and a minor loss of paternal repression of protein-coding genes with the paternal *Igf2r* promoter showing a normal gain of DNA methylation (the *Slc22a2* and *Slc22a3* genes are repressed in the absence of promoter methylation [49]). A direct comparison between the two imprinted clusters is not possible since although *Kcnq1ot1* steady-state levels were unchanged in both the deletion experiments [18], [47], measurements were only made in the first half of the transcript and it is not known if these deletions affected the full-length of *Kcnq1ot1*.

### The *Airn* downstream CGI controls efficient transcription initiation and elongation

Classic mammalian promoter-associated CGIs extend upstream and downstream of the transcription start of the majority of mouse and human genes and these CGIs are considered to have promoter regulatory functions [1]. The promoter region of a CGI is perceived as the region between the 5′ boundary of the CGI and the TSS [50], although none have been subject to deletion at the endogenous locus and analyzed as described here. Recently, evidence has been accumulating that gene regulation acts not only at the step of RNAPII recruitment by the promoter, but also at later steps of transcription elongation and processing [51]–[53]. The data here show that elements located downstream of the transcription start site are required for RNAPII transcription initiation and elongation and also indicate that CGIs can play a different role to that of the upstream promoter.

Reduced *Airn* transcript length could be explained by alternative polyadenylation site choice that is often seen in mammalian genes [54]. The *Airn* ncRNA produces four splice variants, three of which have alternative polyadenylation sites spread over 45 kb (Figure 2) [21]. Although premature polyadenylation could explain progressive *Airn* shortening in *TDRΔ* and *CGIΔ* alleles, we think this unlikely for two reasons. First, the genome tiling array analysis shows *Airn* shortening is gradual and not stepwise, which would be expected from use of alternative polyadenylation sites. Second, the RT-qPCR data indicate that *Airn* shortening on *CGIΔ* alleles occurs within the first 570 bp, which does not contain a known polyadenylation site (http://rulai.cshl.edu/tools/polyadq/polyadq_form.html). Cells with a paternal *TDRΔ* allele showed similar occupancy of the initiating and elongating forms of RNAPII to wildtype cells, except for the 3′ end of *Airn* where elongating RNAPII was reduced. As *Airn* transcription initiation is unchanged in *TDRΔ* cells with the majority of transcripts longer than 68 kb, this indicates the length of *Airn* is subject to regulation after the switch between paused and elongated transcription. In cells with a *CGIΔ* allele however, both initiating and elongating RNAPII were decreased compared to wildtype and *TDRΔ* cells, although ∼60% of wildtype RNA levels were found at the very 5′ end. This indicates that the deletion of the whole CGI affected the ability of the upstream promoter region not only to elongate but also to efficiently initiate *Airn* transcription. The finding that both the TDR and CGI deletions induced progressive *Airn* shortening indicates that cumulative elements distributed throughout the CGI play distinct roles in regulating *Airn* transcription elongation and processivity.

An obvious feature involved in regulating expression of a CGI associated gene is DNA methylation. Gain of methylation was not seen on the paternal *TDRΔ* allele, but up to 70% of DNA methylation was gained on the flanking sequences after paternal CGI deletion. However, *Airn* 5′ levels only showed a moderate change on the *CGIΔ* allele. As methylation levels showed a high variability between different alleles, ranging from 9–100%, it could be possible that hypomethylated alleles are still able to initiate *Airn* transcription as detected by RT-qPCR which specifically analyses *Airn* transcripts, but not by the RNAPII ChIP which might be relatively less sensitive and also suffers from background problems due to increased *Igf2r* levels in the overlap region. We therefore suggest that this gain of methylation and not loss of the CGI, explains the reduction in *Airn* transcription initiation shown by the *CGIΔ* allele. Since most CpG dinucleotides including those in the body of genes, are methylated when they lie outside CGIs [55], it is clear that DNA methylation downstream of promoters does not block transcript elongation of endogenous mammalian genes. Furthermore, as no increase in DNA methylation was observed upon TDR deletion on the paternal allele that also induced shortening of *Airn*, we can exclude DNA methylation as the cause of the length phenotype. Thus, loss of sequences within the CGI and not gain of DNA methylation correlate with loss of full-length *Airn*.

### The *Airn* CGI is required to block DNA methylation on its paternal promoter

Deletion of the TDRs removed the 3′ half of the CGI from the paternal ICE but did not change its unmethylated status. Notably, controls used in these experiments allowed us to observe for the first time, a low level of DNA methylation on the wildtype paternal ICE in two different undifferentiated ES cell lines, that was fully reversible upon differentiation and was also absent in differentiated primary embryonic fibroblasts. Although *Airn* is not expressed in undifferentiated ES cells, the paternal ICE is marked by H3K4me3 [31], [56], which has been shown to block DNMT3L, an essential cofactor for the *de novo* methylation complex, from binding histone H3 [57]. The existence of low-level DNA methylation at the *Airn* promoter in undifferentiated cells despite the presence of H3K4me3 indicates either, that high *Airn* expression induced during differentiation is required in addition to H3K4me3 to fully block DNA methylation or, that DNA methylation modifies a small number of chromosomes in the population that lack H3K4me3 [58]. Deletion of the whole CGI led to a substantial gain of DNA methylation on the paternal allele that was not reversible upon differentiation but was enhanced after removal of the selection cassette. We attribute this enhancement to the longer period in cell culture required to remove the cassette. Thus the CGI deletion shows that one role located in the first half of the island, is to block DNA methylation on the paternal *Airn* promoter that is 177 bp upstream from the deleted sequences. Transgene reporter experiments have been used to show that SP1 transcription factor binding sites protect a CGI from DNA methylation [59]–[62]. Furthermore a high CpG density also correlates with protection from DNA methylation by recruitment of the CpG-binding protein CFP1, which in turn leads to H3K4me3 via recruitment of the SETD1 histone methyltransferase [58]. As the CGI deletion reduces CpG density considerably and removes three predicted SP1 binding sites [21], this may explain the gain of DNA methylation upon deletion of the CGI.

### The 3′ part of the CGI is necessary for the maternally methylated state of the ICE

Deletion of the 3′ half of the CGI that included the TDRs, led to loss of ICE methylation following maternal transmission of the deleted allele. The *Airn*-TDRs are conserved in human and mouse at an organizational level and in their ability to be methylated on the maternal chromosome only [27], [63]. The conservation of TDRs in the ICE may be explained by the preference of the DNMT3A *de novo* methyltransferase for an 8–10 bp periodicity in CpG frequency, that is seen in the 12 known maternally-methylated ICE [64]. Previous experiments using multicopy transgenes randomly inserted in the genome have also identified the *Airn*-TDRs, in particular the three long 172–180 bp monomer repeats, as important for maternal-specific methylation of a hybrid *RSVIgmyc* imprinted transgene [65]. These experiments also demonstrated a role for the TDRs in maintaining the unmethylated state on paternal transmission. The data reported here that deleted the TDRs from the endogenous *Airn* CGI, confirm a role for the TDRs in the methylation of the maternal ICE, but do not demonstrate a role in maintaining the unmethylated state of the paternal ICE. The two overlapping 657 and 890 bp deletions cited above for the *Kcnq1ot1* downstream CGI [18], [47], were not directly tested for their role in the methylation of the maternal ICE. Indirect evidence that indicates no role for these deleted regions comes from the finding that the maternal transmission of the 890 bp deletion did not lead to derepression of *Kcnq1ot1*. Together this would indicate that the *Airn*-TDRs but not the *Kcnq1ot1* TDRs, have a function in methylation of the maternal ICE. However two minor caveats could be considered. First, the two overlapping deletions reported from the *Kcnq1ot1* downstream CGI might not have removed all necessary sequences and second, these two overlapping deletions left a single loxP site at the site of the deletion, which has been reported to attract DNA methylation [33]. In contrast, the *Airn*-TDR deletions reported here placed the remaining single loxP site 2 kb upstream from the deletion and we are now able to assign a specific role for the *Airn*-TDRs in the methylation of the maternal ICE at the endogenous locus.

Together the data presented here show that the CGI lying immediately downstream of the *Airn* transcription start regulates both the epigenetic and transcription state of its upstream promoter. Classically, with the exception of retrotransposons, RNA polymerase II promoters are viewed as lying upstream of the transcription start [66], [67]. In contrast, the majority of CGIs can be seen in Figure 1C to extend downstream of the transcription start, with some located entirely downstream of the transcription start. The importance of CGIs as regulators of gene expression has been emphasised with the advent of genome-wide studies showing CGIs are not only associated with genes showing tissue-specific and inducible expression but are also present in large numbers as orphan CGIs not associated with annotated promoters [1], [2], [68]. The data here identify a role for the downstream *Airn* CGI to regulate its epigenetic state and the production of transcripts expressed at sufficiently high levels and of sufficient length to silence flanking target genes. Future work will determine how this regulation is achieved and if these features are shared by CGIs regulating non-imprinted gene expression.

## Materials and Methods

### Ethics statement

Mice were bred and housed at the Forschungsinstitut für Molekulare Pathologie GmbH, Dr. Bohr-Gasse 7, 1030 Vienna, Austria in strict accordance with national recommendations described in the “IMP/IMBA Common Institutional policy concerning the care and use of live animals” with the permission of the national authorities under Laboratory Animal Facility Permit MA58-0375/2007/4. Blastocyst injections and chimeric mice were prepared under the permit M58/003079/2009/8: Production of Chimeras, Examination of Germline, Examination of Gene Effects in Parents and Successor Generations (Model B). Mouse embryos were obtained after humane killing of pregnant female mice by cervical dislocation by skilled qualified personnel.

### Generation of knock-out ES cells and mice

All targeting vectors were generated from a plasmid with a 6.4 kb 129Sv homology region (chr17:12931344–12937792/NCBI37-mm9). In the *TDRΔ* construct a 692 bp SacII-NsiI fragment (chr17:12934848–12935543) was deleted. The selection cassette (loxP)-(*HSVTk-Neomyocin-SV40*polyA)-(*HSVTk-ThymidineKinase+*polyA)-(loxP) for the ES cell imprinting model and (loxP)-(*Pgk1-Neomycin-Pgk1*polyA)-(loxP) for blastocyst injection was subcloned into the NheI site at chr17:12932836. In the *CGIΔ* construct the 1129 bp deletion (chr17: 12934414–12935543) was created by PCR (primers: TGGAACCCTTCCTTTGCGGAATC - TGCATGAGGGTGCCACACTCCT). The selection cassette: (loxP)-(*Pgk1*-*Neomycin*-*Pgk1*polyA)-(loxP) was inserted at the same position as for *TDRΔ*. Electroporation and neomycin-selection were performed using standard conditions into *S12/+* cells (a D3 feeder-dependent 129 ES line previously modified to carry a SNP in *Igf2r* exon 12 [31]) for the ES cell imprinting model experiments and into the feeder-dependent BL6/129 intraspecies A9 ES cell line for blastocyst injection. The selection cassette in the ES cells used for the ES cell imprinting model was removed by electroporation of the pMC-Cre plasmid leaving a single loxP site 2 kb upstream of each deletion. One A9 ES cell clone carrying the *TDRΔ+cas* allele was injected into C57BL/6J blastocysts and transferred into pseudo-pregnant recipient mice and one chimeric male mouse was obtained who transmitted the *TDRΔ+cas* allele. The selection cassette was removed by crossing *TDRΔ+cas* males with MORE-Cre females. Heterozygous *TDRΔ* mice were mated with wildtype FVB or FVB with a *Thp* allele and embryos were isolated at 12.5 dpc or 13.5 dpc. Visceral yolk sacs were isolated as described in [29].

### ES cell culture and differentiation

ES cells were grown on irradiated primary mouse embryonic fibroblasts using standard conditions and differentiation induced by feeder-depletion, LIF-withdrawal and 0.27 µM retinoic acid.

### RNA and DNA analysis

RNA was isolated using TRIreagent and was DNaseI treated prior to reverse transcription. Realtime qPCR for Taqman assays was as described [21]. SybrGreen assays used 100 nM primers and cycling conditions: 5 min 95°C, 40 cycles: 15 sec 95°C+1 min 60–65°C. Allele-specific qPCR was as described [31] with 5 mM MgCl_2_. The assay specificity was improved by a mismatch in the primer body. See for primers and probes. All assays were normalised to CyclophilinA. DNA isolation and blots were performed using standard techniques and ImageJ quantified signal intensities. For some blots the contrast was linearly enhanced with Adobe Photoshop. RNA hybridization to genome tiling array was performed by Source BioScience LifeSciences, Berlin, as described [69]. The data were Tukey bi-weight normalised before analysis. Relative signal intensities (normalised to the average signal in the region) of overlapping windows of 9 tiles were averaged and each displayed data point is the average of 20 windows, the standard deviation is displayed as error bars. Two pseudogenes in the region, Au76 and LA41, were removed from the analysis. RNA sequencing:1 µg of total RNA was treated with the RiboZero kit (Epicentre) and two strand-specific RNA-Seq libraries prepared using the ScripSeq kit and two compatible barcodes (Epicentre) according to the manufacturer's protocol. Sequencing and read alignment to the mouse genome (mm9) was as described [70]. The region shown in Figure 6A was divided into non-overlapping 3.2 kb windows, reads mapping to the forward or reverse strand in these windows were counted and the log2 ratio of these counts was calculated and plotted. Windows overlapping *Igf2r* exons and the *Au76* and the *LA41* pseudogenes were removed from the plot.

### Chromatin immunoprecipitation

Preparation of soluble chromatin and chromatin immunoprecipitation assays were carried out as described [71]. 25 µg of sonicated chromatin were diluted 10-fold and precipitated overnight with the following antibodies: Phospho RNA Pol II (S5) (Bethyl Laboratories A300-655A), Phospho RNA Pol II (S2) (Bethyl Laboratories A300-654A) or rabbit IgG (Invitrogen 10500C) as control. Chromatin antibody complexes were isolated using Protein A magnetic beads (Dynabeads). The extracted DNA was then used for qPCR as described above. A 1∶20 dilution of input DNA was assayed.

### Bisulfite sequencing

1 µg genomic DNA from undifferentiated ES cells grown on feeder cells carrying a homozygous ICE deletion or 1 µg genomic DNA from 12.5–13.5 dpc embryos was RNaseA treated, EcoRI digested and Bisulfite converted using the EpiTect Bisulfite Kit (Qiagen). PCR amplification used JumpStart Taq DNA Polymerase (Sigma), primers: DMR2-F4 (GGGGAATTGAGGTAAGTTAGGGTTTT) with DMR2-R4 (TCTTATAACCCAAAAATCTTCACCCTAAC) for wt alleles or DMR2-R9 (AACACCTTCATATACCCCTAAACAC) for *TDRΔ* and *CGIΔ* alleles [8], cycle conditions: 94°C 1 min, 40 cycles of 94°C 1 min, 60°C 1 min, 72°C 1 min then 72°C 5 min. PCR fragments were gel-purified, subcloned and plasmid DNA from single colonies sequenced using standard primers. Analysis and sequence quality control used BiQAnalyzer and standard settings [72].

### CpG island and transcription start site analysis

Each CGI (UCSC Genome Browser, mm9) with flanking regions (50% of the CGI length upstream and downstream) was divided into 100 equal-sized bins (i.e., parts) and the number of RefSeq genes (USCS mm9) was calculated with a transcription start site in each bin. Bins were summed for all CGIs and plotted using Microsoft Excel.

### Statistical analysis

For qPCRs an unpaired t-test was performed using www.graphpad.com/quickcalcs/.

## Supporting Information

Figure S1 Generation of *TDRΔ* ES cells. (A) Targeting strategy. Top: wildtype (wt) allele containing the *Airn* promoter; below: targeting vector that deletes the TDRs (grey bar) lying in the 3′ part of the CGI (grey bar) with homology regions indicated. A floxed selection cassette (*HSV-Tk* promoter-*neomycin resistance (neo)*-*SV40* polyadenylation (*pA*) signal and *HSV-Tk* promoter-*HSVTk* gene-*HSVTk pA*) was inserted into the NheI site in *Igf2r* intron 3. Homologous recombination created the *TDRΔ+cas* allele. Transient transfection with CRE-recombinase plasmid created the *TDRΔ* allele. Note the remaining loxP site in the *TDRΔ* allele lies 1.3 kb upstream of the *Airn* promoter (black triangle). White boxes: *Igf2r* exons. Grey bars: DNA blot probes. Enzymes: E: EcoRI, Bs: BsrGI, X: XbaI, Nh: NheI, ScII: SacII, Ns: NsiI, Bm: BmiI. (B) Left: DNA blot genotyping for homologous recombination. Genomic DNA from two independently-targeted ES cells (*S12/TDRΔ+cas*-1,-2) and their parental ES cell line (*S12/+*) was digested with EcoRI and hybridised with probe *Airn*T. The 5.5 kb band indicates homologous recombination, creating the *TDRΔ+cas* allele. Right: DNA blot genotyping for loss of selection cassette. Genomic DNA from targeted cells before (*S12/TDRΔ+cas*-1,-2) and after (*S12/TDRΔ*-1A,-1B,-2A,-2B) CRE-expression and the original parental ES cell line (*S12/+*) was digested with BsrGI and hybridised with probe MEi. Subclones A and B were derived from the independently targeted clones 1 and 2. Loss of the 8.4 kb band and gain of the 5.4 kb band indicates CRE-mediated recombination. (C) Genomic locus and DNA blot strategy to analyse the parental specificity of targeting. E: EcoRI, M: MluI. White boxes: *Igf2r* exons. Grey bar: DNA blot probe. (D) DNA blot of *S12/+* parental ES cells and ES cells carrying a *TDRΔ* allele. Genomic DNA was digested using EcoRI and MluI and hybridised using probe *Airn*T. The loss of the 5 kb and gain of the 4.3 kb band in *S12/TDRΔ* ES cells shows the paternal allele was targeted.
(JPG)

Figure S2 Generation of *TDRΔ* mouse. (A) Targeting strategy: As in Figure S1A but using a different selection cassette (*Pgk1* promoter driving a *neomycin resistance* gene (*neo*) stopped by a *Pgk1* polyadenylation signal (*pA*); flanked on both sides by loxP sites). ES cells with a *TDRΔ+cas* allele were injected into blastocysts, mice carrying a targeted allele were identified and the selection cassette was removed by mating with a MORE-CRE expressing mouse (see Methods). Note the remaining loxP site in the *TDRΔ* allele lies 1.3 kb upstream of the *Airn* promoter (black triange). (B) DNA blots of ES cell DNA confirm homologous recombination with an external (*Airn*T) and an internal (MEi) probe. Further details as in Figure S1B. (C) DNA blots of mouse tail DNA confirming germline transmission of the *TDRΔ+cas* allele and successful removal of the selection cassette (5.4 kb band) to generate the *TDRΔ* allele. Details as in Figure S1B.
(JPG)

Figure S3 Expression of imprinted genes in *TDRΔ* mice - additional data. (A) qPCR of unspliced *Airn* in VYS confirms a significant decrease in *Airn* steady-state levels at the 3′ end upon paternal transmission of *TDRΔ* as seen in ES cells and embryos. Details as in Figure 3C. (B) - (D) qPCR analysis of *Igf2r*, *Slc22a2* and *Slc22a3* shows a modest but not consistently significant increase in expression of the paternal allele in the VYS of mice carrying a paternally transmitted *TDRΔ* allele. Details as in Figure 4D. (E) qPCR of unspliced *Airn* in embryos at three positions along its length, shows that maternal transmission of the *TDRΔ* allele leads to upregulation of *Airn* from the maternal allele with a similar length phenotype as observed after paternal transmission of the *TDRΔ* allele. Details as in Figure 3C. *+/+* and *TDRΔ/+* embryos were compared using an unpaired t-test. As only one *TDRΔ/Thp* embryo was obtained, no error bars are plotted and no statistical comparison was performed with *+/Thp* embryos. (F) qPCR analysis of *Igf2r* in embryos reveals a significant reduction of *Igf2r* levels after maternal transmission of the *TDRΔ* allele showing that expression of the *TDRΔ-Airn* from the maternal chromosome leads to repression of the maternal *Igf2r* promoter. Details as in (E). (G) qPCR of unspliced *Airn* in VYS show *Airn* expression after maternal transmission as observed in embryos of the same genotypes. Details as in (E). (H)–(J) qPCR analysis of *Igf2r*, *Slc22a2* and *Slc22a3* shows a significant reduction of steady-state levels after maternal transmission of the *TDRΔ* allele showing that expression of the *TDRΔ-Airn* from the maternal chromosome leads to repression of the maternal alleles of these genes. Details as in (E).
(JPG)

Figure S4 Absence of TDRs compromises imprinted expression of *Igf2r*; additional differentiation sets. As in Figure 4A, 4B for two further differentiation sets.
(JPG)

Figure S5 Tandem direct repeats are required for the regulation of ICE DNA methylation; additional differentiation sets and controls. (A) As in Figure 5A for two further differentiation sets. (B) Overview of the region analysed by bisulfite sequencing. T1,T2,T3: *Airn* transcription start sites. MluI: MluI site used to assay DNA methylation by DNA blot. Arrows: primers used to amplify bisulfite converted DNA. Horizontal grey lines: *CGIΔ* and *TDRΔ*. (C) Bisulfite analysis of undifferentiated *S12/+*, *R2Δ/+*, *+/R2Δ* ES cells and *Thp/+* and *+/Thp* primary (p) MEFs. The primers are shown below the genotype. Details as in Figure 5B. Note that the *R2Δ* and *Thp* alleles are deleted for the ICE, thus specific amplification of the wildtype allele is achieved. In undifferentiated *S12/+* ES cells both parental alleles were amplified and approximately half of the clones shows high, the other half low level of DNA methylation. In *R2Δ/+* ES cells only the paternal allele was amplified and confirms a low level of DNA methylation present in undifferentiated ES cells on the paternal allele. In *+/R2Δ* ES cells only the maternal allele was amplified and all sequenced clones show a high level of DNA methylation. In *Thp/+* pMEFs only the paternal allele was amplified and shows complete absence of DNA methylation. In *+/Thp* pMEFs only the maternal allele was amplified and shows in 11/12 sequences 100% DNA methylation. (D) Bisulfite analysis of undifferentiated *S12/TDRΔ*-2A ES cells. Details as in Figure 5B. (E) Plot showing percent methylation level for individually sequenced clones for *S12/+*, *R2Δ/+* (same plot as shown in Figure 5C), *+/R2Δ* ES cells and *Thp/+*, *+/Thp* pMEFs. Details as in Figure 5C. (F) Bisulfite analysis and plot showing percent methylation level for individually sequenced clones of *TDRΔ/+* and *+/+* embryos. For *TDRΔ/+* only the *TDRΔ* allele, for *+/+* both parental alleles were sequenced. Details as in Figure 5B, 5C.
(JPG)

Figure S6 Generation of *CGIΔ* ES cells. (A) Targeting strategy for generating *CGIΔ* ES cells. The CGI downstream of *Airn* was deleted in the targeting vector, from 29 bp downstream of the MluI site until the NsiI site. The same selection cassette as in Figure S2A was inserted into the NheI site in *Igf2r* intron 3. Further details as in Figure S1A. Note that the remaining loxP site in the *CGIΔ* allele lies 1.3 kb upstream of the *Airn* promoter (black triangle). (B) Left: Genotyping by DNA blotting of ES cells carrying a *CGIΔ+cas* allele. Gain of the 5.1 kb band is indicative of homologous recombination. Right: Genotyping by DNA blotting of ES cells carrying a *CGIΔ+cas* or a *CGIΔ* allele and the original parental ES cell line (*S12/+*). Loss of the 6.8 kb band and gain of the 5.0 kb band is indicative of CRE-mediated recombination. Further details as in Figure S1B. (C) Left: Overview of the genomic locus and DNA blot strategy to analyse the parental targeting of *CGIΔ+cas* ES cells. Right: DNA blots of *S12/+* parental ES cells and ES cells carrying a *CGIΔ+cas* allele digested with EcoRI and the methyl-sensitive MluI enzyme. Further details as in Figure S1C and S1D. The presence of a strong 3.9 kb band in combination with an unchanged 6.2 kb band in *S12/CGIΔ* ES cells is indicative of a paternal targeting event. Although the weak 5.0 kb band in the *S12/CGIΔ* ES cells could be explained by feeder cell contamination, further analysis presented in Figure 7A and 7B demonstrates that this band originates from a gain of DNA methylation on the paternally targeted *CGIΔ* allele.
(JPG)

Figure S7 Deletion of the *Airn* CGI results in biallelic expression of *Igf2r*; additional differentiation sets. As Figure 6D for two further differentiation sets. Lower panel in A: *methylated fragment in d0 cells originating from feeder-cells.
(JPG)

Figure S8 The methylation-free state of the paternal ICE depends on the CGI; additional differentiation sets. As in Figure 7B for two further differentiation sets. Note that the top blot was hybridised with probe *Airn*T, whereas the bottom blot and the blot in Figure 7B were hybridised with probe MEi. The white lines in the bottom blot indicate that the order of the samples was changed electronically, as they were loaded in a different order but all on the same gel.
(JPG)

Table S1 Primers and probes for qPCR assays, PCR assays, and DNA blots.
(DOC)
