# Supplementary figures and images for: A Downstream CpG Island Controls Transcript Initiation and Elongation and the Methylation State of the Imprinted Airn Macro ncRNA Promoter
Source: PLoS Genet. 2012 Mar 1;8(3):e1002540. doi: 10.1371/journal.pgen.1002540 (PMC3291542; doi:10.1371/journal.pgen.1002540)

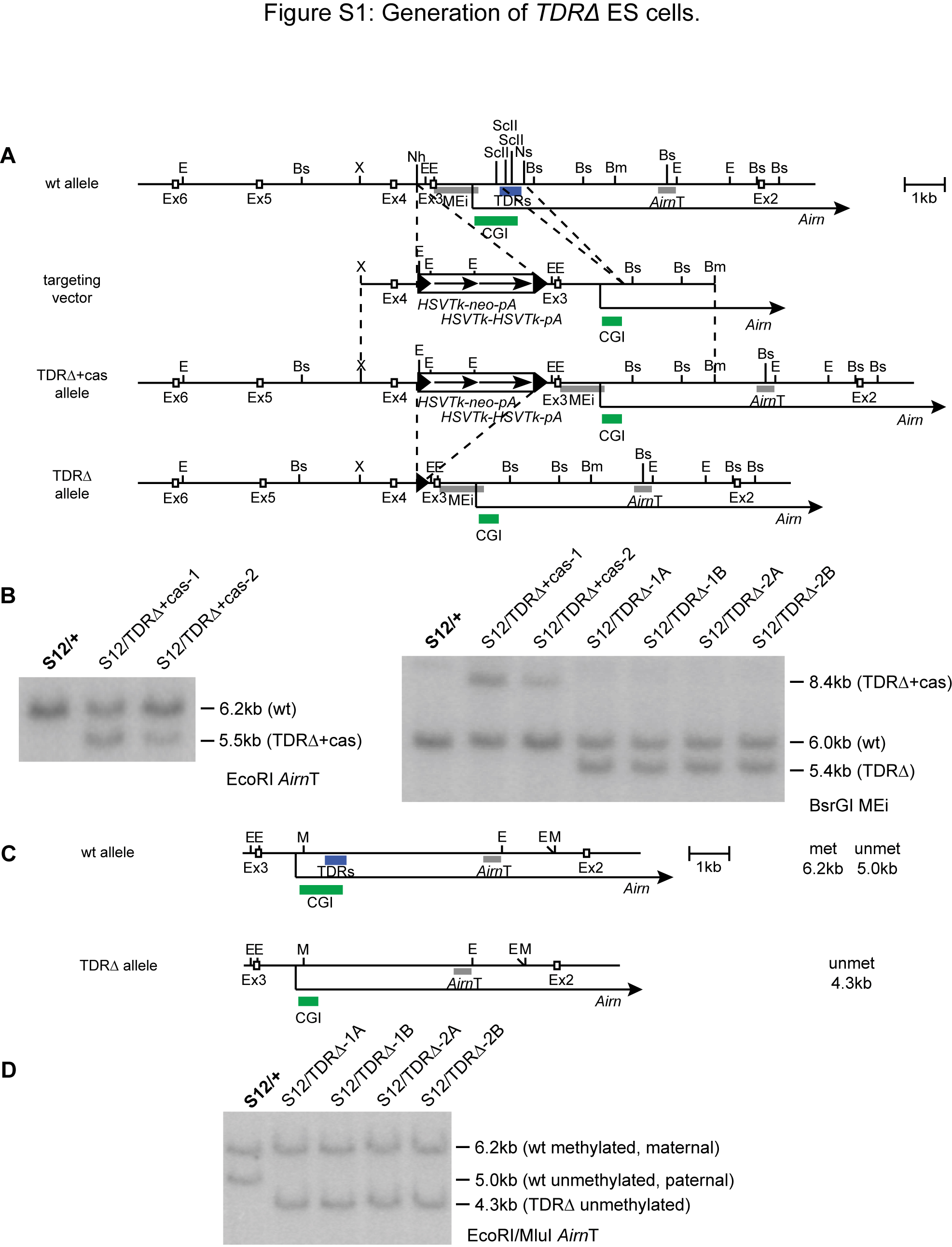

Supplement: Figure S1 — Generation of TDRΔ ES cells. (A) Targeting strategy. Top: wildtype (wt) allele containing the Airn promoter; below: targeting vector that deletes the TDRs (grey bar) lying in the 3′ part of the CGI (grey bar) with homology regions indicated. A floxed selection cassette (HSV-Tk promoter-neomycin resistance (neo)-SV40 polyadenylation (pA) signal and HSV-Tk promoter-HSVTk gene-HSVTk pA) was inserted into the NheI site in Igf2r intron 3. Homologous recombination created the TDRΔ+cas allele. Transient transfection with CRE-recombinase plasmid created the TDRΔ allele. Note the remaining loxP site in the TDRΔ allele lies 1.3 kb upstream of the Airn promoter (black triangle). White boxes: Igf2r exons. Grey bars: DNA blot probes. Enzymes: E: EcoRI, Bs: BsrGI, X: XbaI, Nh: NheI, ScII: SacII, Ns: NsiI, Bm: BmiI. (B) Left: DNA blot genotyping for homologous recombination. Genomic DNA from two independently-targeted ES cells (S12/TDRΔ+cas-1,-2) and their parental ES cell line (S12/+) was digested with EcoRI and hybridised with probe AirnT. The 5.5 kb band indicates homologous recombination, creating the TDRΔ+cas allele. Right: DNA blot genotyping for loss of selection cassette. Genomic DNA from targeted cells before (S12/TDRΔ+cas-1,-2) and after (S12/TDRΔ-1A,-1B,-2A,-2B) CRE-expression and the original parental ES cell line (S12/+) was digested with BsrGI and hybridised with probe MEi. Subclones A and B were derived from the independently targeted clones 1 and 2. Loss of the 8.4 kb band and gain of the 5.4 kb band indicates CRE-mediated recombination. (C) Genomic locus and DNA blot strategy to analyse the parental specificity of targeting. E: EcoRI, M: MluI. White boxes: Igf2r exons. Grey bar: DNA blot probe. (D) DNA blot of S12/+ parental ES cells and ES cells carrying a TDRΔ allele. Genomic DNA was digested using EcoRI and MluI and hybridised using probe AirnT. The loss of the 5 kb and gain of the 4.3 kb band in S12/TDRΔ ES cells shows the paternal allele was targeted. (JPG) [file pgen.1002540.s001.jpg]

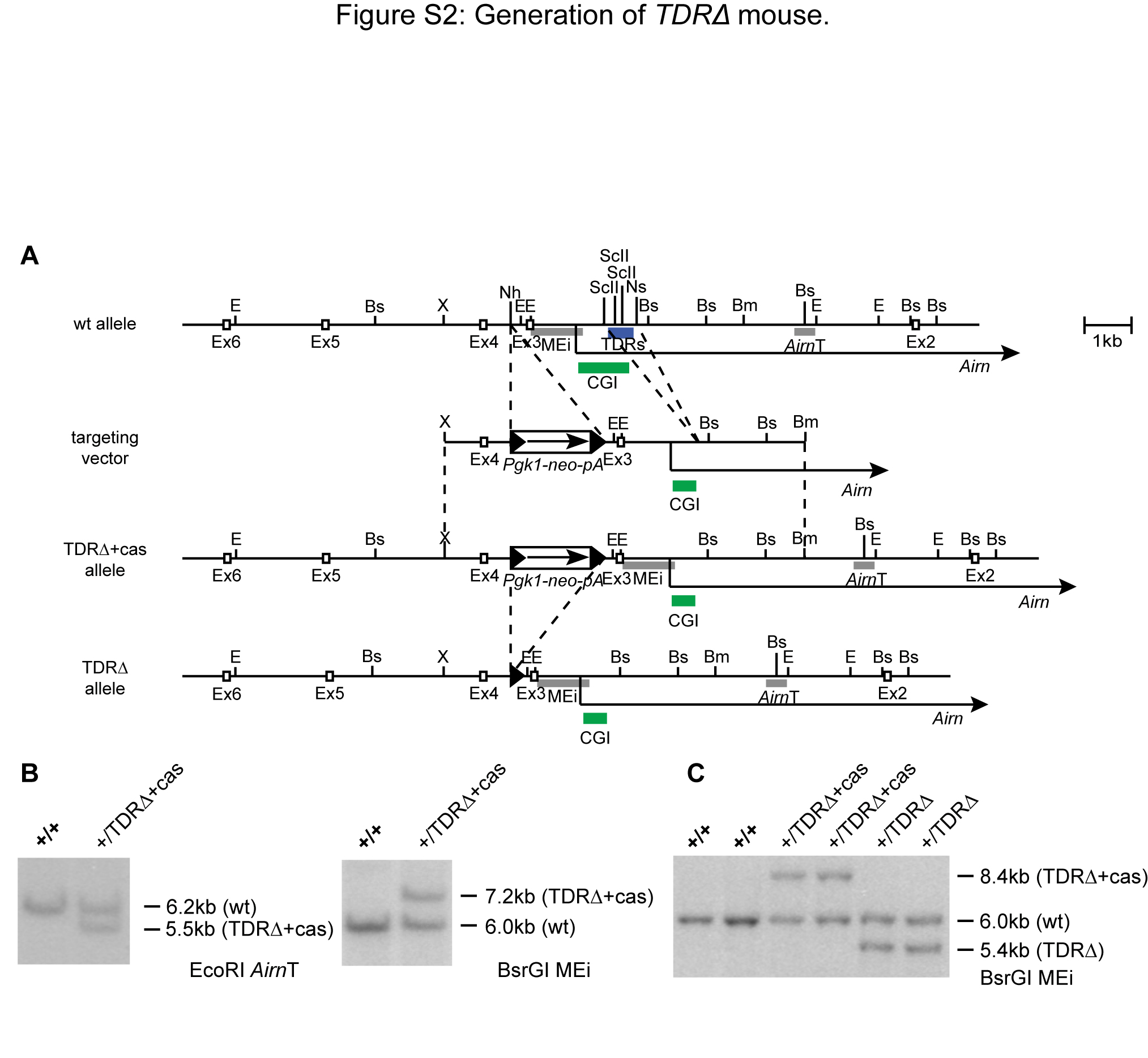

Supplement: Figure S2 — Generation of TDRΔ mouse. (A) Targeting strategy: As in Figure S1A but using a different selection cassette (Pgk1 promoter driving a neomycin resistance gene (neo) stopped by a Pgk1 polyadenylation signal (pA); flanked on both sides by loxP sites). ES cells with a TDRΔ+cas allele were injected into blastocysts, mice carrying a targeted allele were identified and the selection cassette was removed by mating with a MORE-CRE expressing mouse (see Methods). Note the remaining loxP site in the TDRΔ allele lies 1.3 kb upstream of the Airn promoter (black triange). (B) DNA blots of ES cell DNA confirm homologous recombination with an external (AirnT) and an internal (MEi) probe. Further details as in Figure S1B. (C) DNA blots of mouse tail DNA confirming germline transmission of the TDRΔ+cas allele and successful removal of the selection cassette (5.4 kb band) to generate the TDRΔ allele. Details as in Figure S1B. (JPG) [file pgen.1002540.s002.jpg]

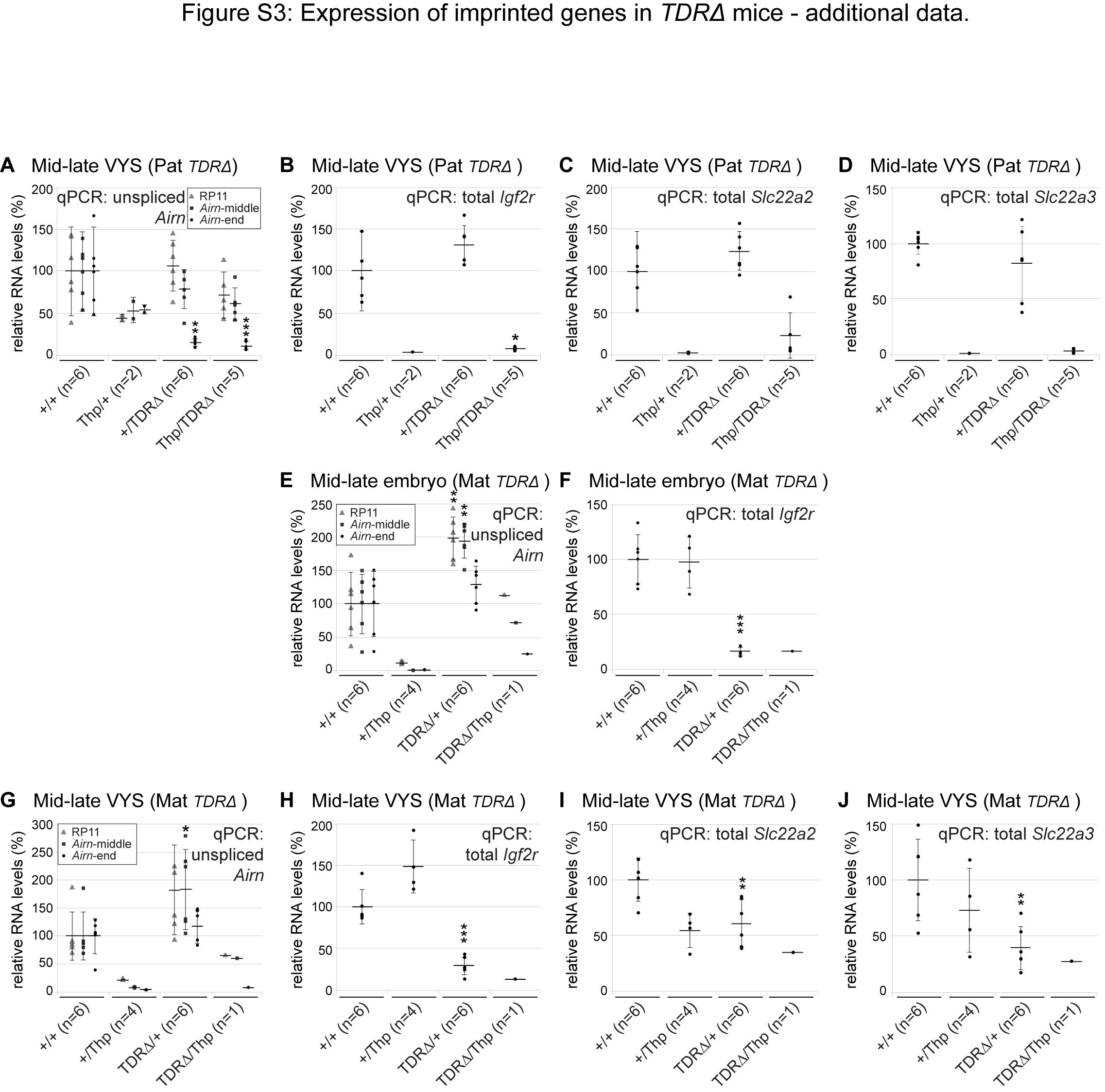

Supplement: Figure S3 — Expression of imprinted genes in TDRΔ mice - additional data. (A) qPCR of unspliced Airn in VYS confirms a significant decrease in Airn steady-state levels at the 3′ end upon paternal transmission of TDRΔ as seen in ES cells and embryos. Details as in Figure 3C. (B) - (D) qPCR analysis of Igf2r, Slc22a2 and Slc22a3 shows a modest but not consistently significant increase in expression of the paternal allele in the VYS of mice carrying a paternally transmitted TDRΔ allele. Details as in Figure 4D. (E) qPCR of unspliced Airn in embryos at three positions along its length, shows that maternal transmission of the TDRΔ allele leads to upregulation of Airn from the maternal allele with a similar length phenotype as observed after paternal transmission of the TDRΔ allele. Details as in Figure 3C. +/+ and TDRΔ/+ embryos were compared using an unpaired t-test. As only one TDRΔ/Thp embryo was obtained, no error bars are plotted and no statistical comparison was performed with +/Thp embryos. (F) qPCR analysis of Igf2r in embryos reveals a significant reduction of Igf2r levels after maternal transmission of the TDRΔ allele showing that expression of the TDRΔ-Airn from the maternal chromosome leads to repression of the maternal Igf2r promoter. Details as in (E). (G) qPCR of unspliced Airn in VYS show Airn expression after maternal transmission as observed in embryos of the same genotypes. Details as in (E). (H)–(J) qPCR analysis of Igf2r, Slc22a2 and Slc22a3 shows a significant reduction of steady-state levels after maternal transmission of the TDRΔ allele showing that expression of the TDRΔ-Airn from the maternal chromosome leads to repression of the maternal alleles of these genes. Details as in (E). (JPG) [file pgen.1002540.s003.jpg]

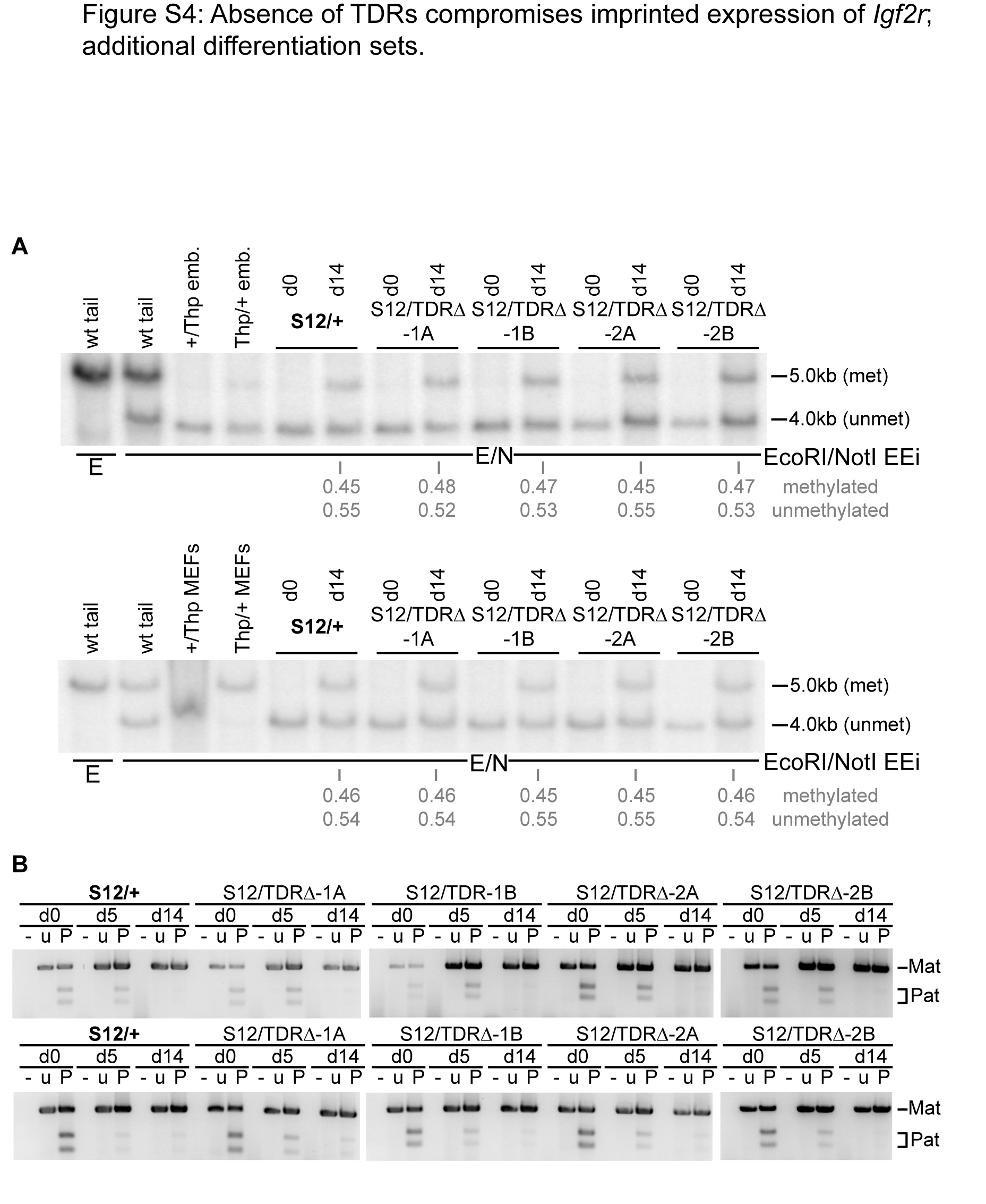

Supplement: Figure S4 — Absence of TDRs compromises imprinted expression of Igf2r; additional differentiation sets. As in Figure 4A, 4B for two further differentiation sets. (JPG) [file pgen.1002540.s004.jpg]

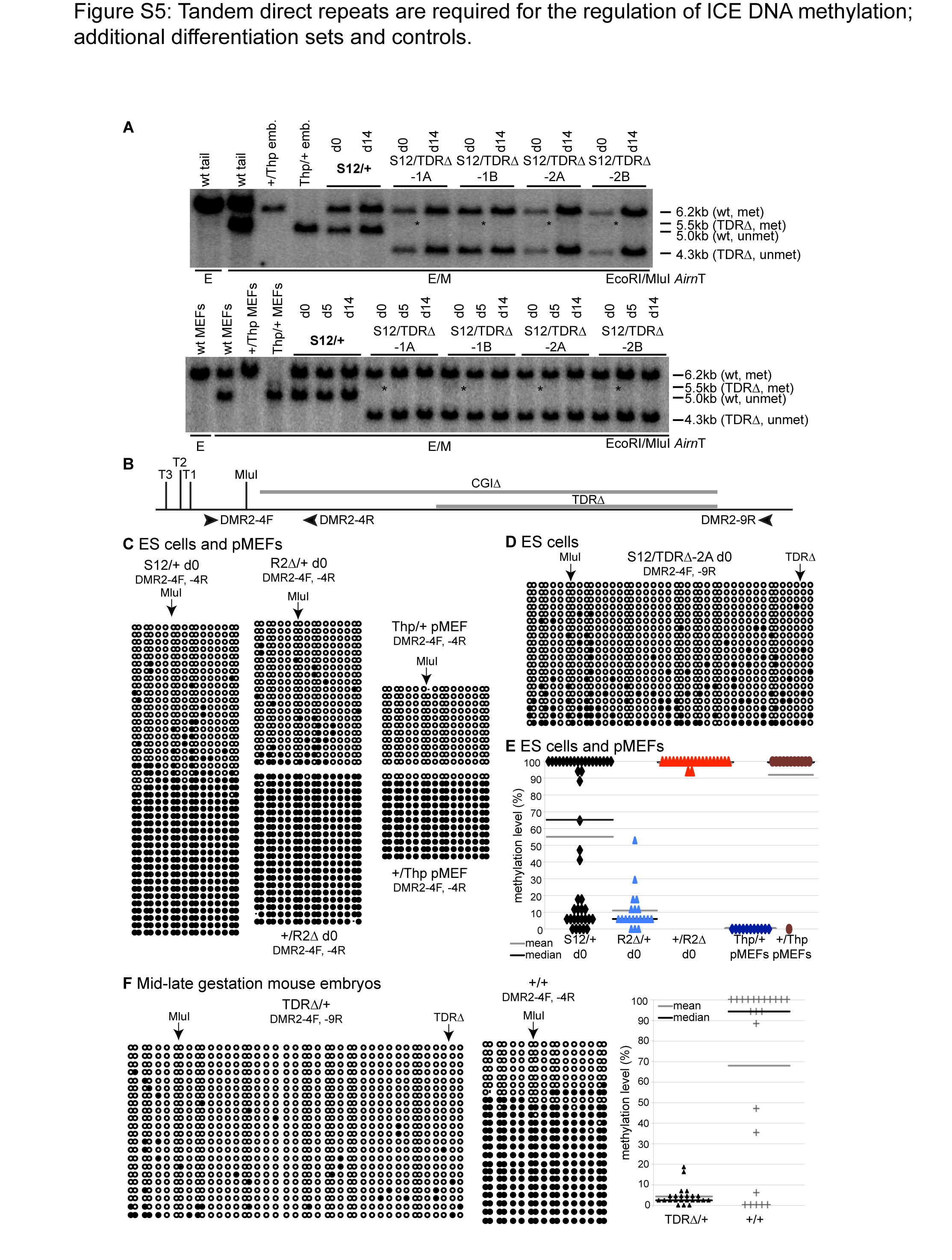

Supplement: Figure S5 — Tandem direct repeats are required for the regulation of ICE DNA methylation; additional differentiation sets and controls. (A) As in Figure 5A for two further differentiation sets. (B) Overview of the region analysed by bisulfite sequencing. T1,T2,T3: Airn transcription start sites. MluI: MluI site used to assay DNA methylation by DNA blot. Arrows: primers used to amplify bisulfite converted DNA. Horizontal grey lines: CGIΔ and TDRΔ. (C) Bisulfite analysis of undifferentiated S12/+, R2Δ/+, +/R2Δ ES cells and Thp/+ and +/Thp primary (p) MEFs. The primers are shown below the genotype. Details as in Figure 5B. Note that the R2Δ and Thp alleles are deleted for the ICE, thus specific amplification of the wildtype allele is achieved. In undifferentiated S12/+ ES cells both parental alleles were amplified and approximately half of the clones shows high, the other half low level of DNA methylation. In R2Δ/+ ES cells only the paternal allele was amplified and confirms a low level of DNA methylation present in undifferentiated ES cells on the paternal allele. In +/R2Δ ES cells only the maternal allele was amplified and all sequenced clones show a high level of DNA methylation. In Thp/+ pMEFs only the paternal allele was amplified and shows complete absence of DNA methylation. In +/Thp pMEFs only the maternal allele was amplified and shows in 11/12 sequences 100% DNA methylation. (D) Bisulfite analysis of undifferentiated S12/TDRΔ-2A ES cells. Details as in Figure 5B. (E) Plot showing percent methylation level for individually sequenced clones for S12/+, R2Δ/+ (same plot as shown in Figure 5C), +/R2Δ ES cells and Thp/+, +/Thp pMEFs. Details as in Figure 5C. (F) Bisulfite analysis and plot showing percent methylation level for individually sequenced clones of TDRΔ/+ and +/+ embryos. For TDRΔ/+ only the TDRΔ allele, for +/+ both parental alleles were sequenced. Details as in Figure 5B, 5C. (JPG) [file pgen.1002540.s005.jpg]

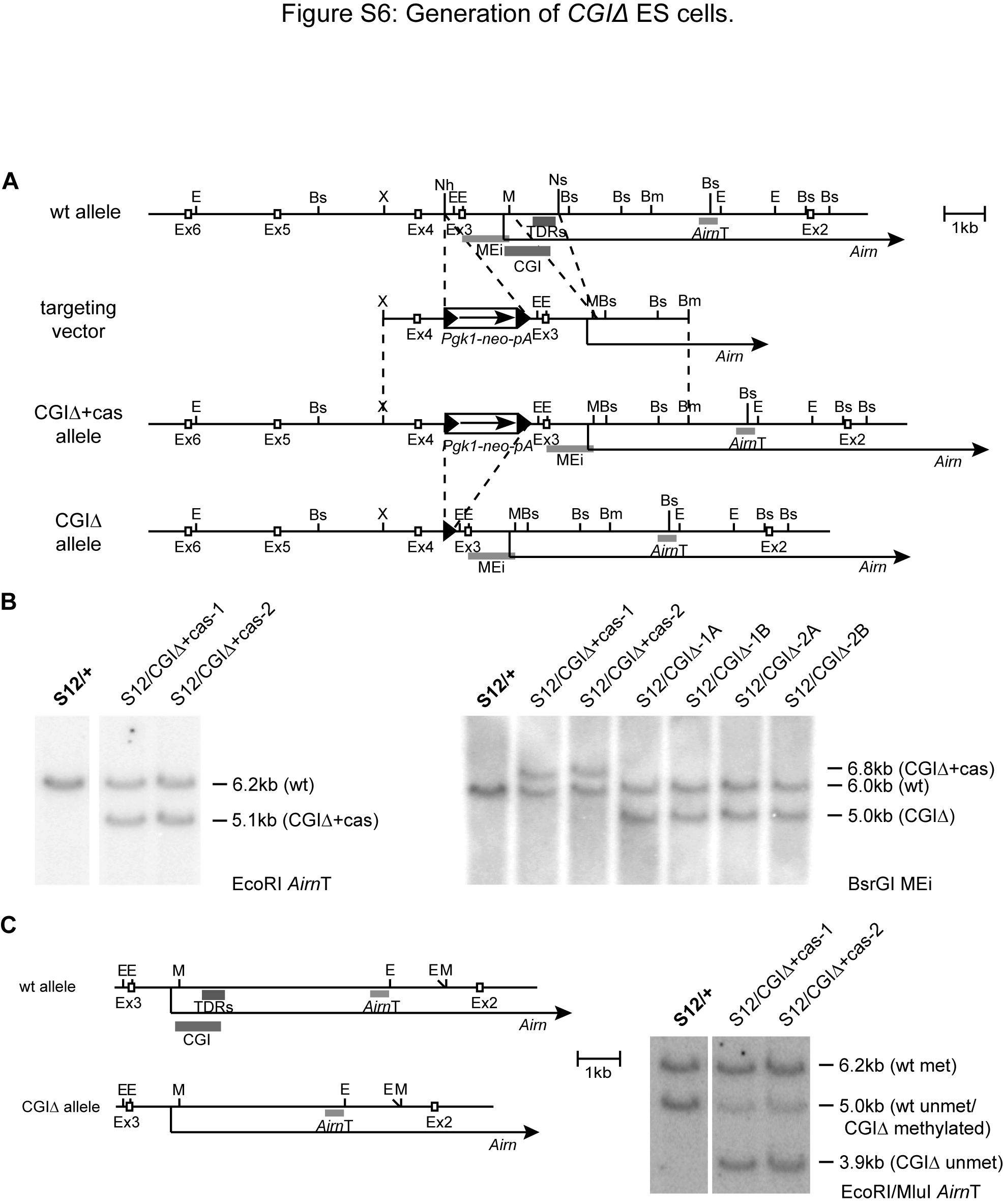

Supplement: Figure S6 — Generation of CGIΔ ES cells. (A) Targeting strategy for generating CGIΔ ES cells. The CGI downstream of Airn was deleted in the targeting vector, from 29 bp downstream of the MluI site until the NsiI site. The same selection cassette as in Figure S2A was inserted into the NheI site in Igf2r intron 3. Further details as in Figure S1A. Note that the remaining loxP site in the CGIΔ allele lies 1.3 kb upstream of the Airn promoter (black triangle). (B) Left: Genotyping by DNA blotting of ES cells carrying a CGIΔ+cas allele. Gain of the 5.1 kb band is indicative of homologous recombination. Right: Genotyping by DNA blotting of ES cells carrying a CGIΔ+cas or a CGIΔ allele and the original parental ES cell line (S12/+). Loss of the 6.8 kb band and gain of the 5.0 kb band is indicative of CRE-mediated recombination. Further details as in Figure S1B. (C) Left: Overview of the genomic locus and DNA blot strategy to analyse the parental targeting of CGIΔ+cas ES cells. Right: DNA blots of S12/+ parental ES cells and ES cells carrying a CGIΔ+cas allele digested with EcoRI and the methyl-sensitive MluI enzyme. Further details as in Figure S1C and S1D. The presence of a strong 3.9 kb band in combination with an unchanged 6.2 kb band in S12/CGIΔ ES cells is indicative of a paternal targeting event. Although the weak 5.0 kb band in the S12/CGIΔ ES cells could be explained by feeder cell contamination, further analysis presented in Figure 7A and 7B demonstrates that this band originates from a gain of DNA methylation on the paternally targeted CGIΔ allele. (JPG) [file pgen.1002540.s006.jpg]

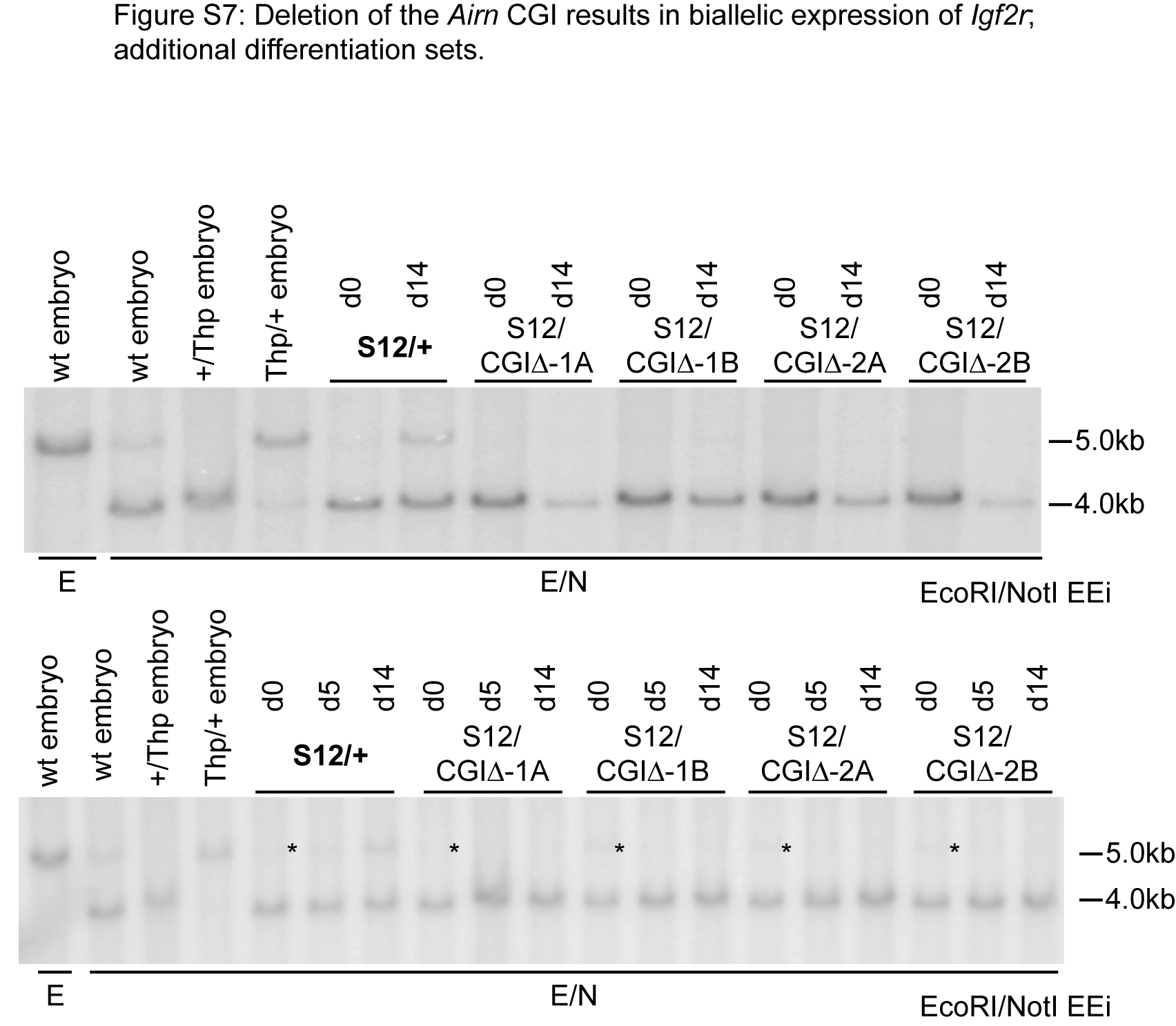

Supplement: Figure S7 — Deletion of the Airn CGI results in biallelic expression of Igf2r; additional differentiation sets. As Figure 6D for two further differentiation sets. Lower panel in A: *methylated fragment in d0 cells originating from feeder-cells. (JPG) [file pgen.1002540.s007.jpg]

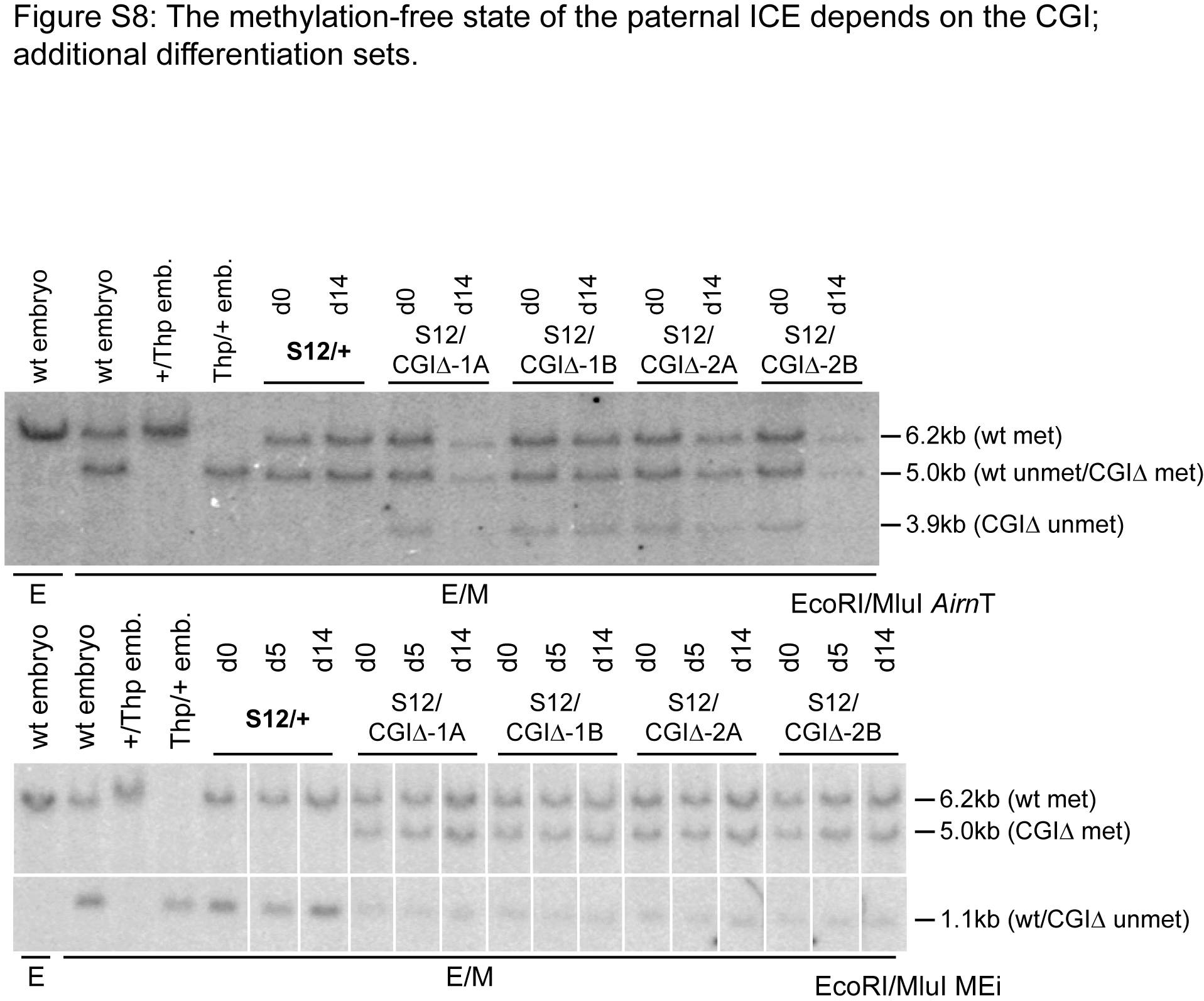

Supplement: Figure S8 — The methylation-free state of the paternal ICE depends on the CGI; additional differentiation sets. As in Figure 7B for two further differentiation sets. Note that the top blot was hybridised with probe AirnT, whereas the bottom blot and the blot in Figure 7B were hybridised with probe MEi. The white lines in the bottom blot indicate that the order of the samples was changed electronically, as they were loaded in a different order but all on the same gel. (JPG) [file pgen.1002540.s008.jpg]
