# Supplementary material for: A Downstream CpG Island Controls Transcript Initiation and Elongation and the Methylation State of the Imprinted Airn Macro ncRNA Promoter
Source: PLoS Genet. 2012 Mar 1;8(3):e1002540. doi: 10.1371/journal.pgen.1002540 (PMC3291542; doi:10.1371/journal.pgen.1002540)
Supplement: Table S1 — Primers and probes for qPCR assays, PCR assays, and DNA blots. (DOC) [file pgen.1002540.s009.doc]

**SUPPORTING TABLE S1.**

**Table S1: Primers and probes for qPCR assays, PCR assays and DNA blots.**

Start/stop: sequence coordinates from NCBI37/mm9.

| **Assay** | **Name** | **Sequence** | **Start (bp)** | **Stop (bp)** | **Assay detection -position relative to *Airn* T1** | **Chr./Acc. number** |
| --- | --- | --- | --- | --- | --- | --- |
| **qPCR assays** | | | | | | |
| ***Airn*-132** | *Airn*-132-F | GAACTGCCAGTGACAAGTGGTAAT | 12927906 | 12927929 | -6.3kb | 17 |
|  | *Airn*-132-P | ^FAM-^TCAGGTTTCTCATGCAGGGTTACAGCAAC^-TAMRA^ | 12927876 | 12927904 |  |  |
|  | *Airn*-132-R | GCACTGTTGCAGAAGTTTATAACCAA | 12927844 | 12927869 |  |  |
| ***Airn*-**  **START** | START-F1 | GCAGCAAGAAGCACAGCAC | 12934237 | 12934255 | -1-39bp | 17 |
|  | START-R1 | GATGTCTGCGTGGTAACTGG | 12934258 | 12934277 |  |  |
| **RP11** | FP1 | AAGCACAGCACCGCCAGT | 12934245 | 12934262 | 7-154bp | 17 |
|  | AS | ^FAM-^CCACGCAGACATC^-MGB^ | 12934265 | 12934277 |  |  |
|  | RP11 | TCCTCTAACGCGTGGAATCC | 12934373 | 12934392 |  |  |
| ***Airn*-124** | *Airn*-124-F | AGAGGTCGAGGGTACGGATATG | 12935917 | 12935938 | 1.6-1.7kb | 17 |
|  | *Airn*-124-P | ^FAM-^CCGGACAGATGGCCCATCTTCCA^-TAMRA^ | 12935893 | 12935915 |  |  |
|  | *Airn*-124-R | TGTCTGAGCTGCAGACAATGG | 12935866 | 12935886 |  |  |
| ***AirnT3*** | *Airn*T3TQF | CCCTAGGAAGGCACAGATGC | 12938294 | 12938313 | 4kb | 17 |
|  | *Airn*T3 | ^FAM-^CCGCTTCCAGCAGCTGTTACATCTAGTGC^-TAMRA^ | 12938264 | 12938292 |  |  |
|  | *Airn*T3TQR | ACAGCGATCCTCCAGAAGAGTG | 12938241 | 12938262 |  |  |
| ***Airn*-117** | *Airn*-117-F | GCCATACTTAAAAGAGCCAAATGTG | 12942788 | 12942812 | 8.5kb | 17 |
|  | *Airn*-117-P | ^FAM-^TGGGAATGGGTGGGAGCTGGCT^-TAMRA^ | 12942760 | 12942781 |  |  |
|  | *Airn*-117-R | TGCTGGGATAAACTTACCCAGAA | 12942726 | 12942748 |  |  |
| ***Airn*-**  **SV1a**  **(RP6)** | FP1 | AAGCACAGCACCGCCAGT | 12934245 | 12934262 | 38kb | 17 |
|  | AS | ^FAM^-CCACGCAGACATC^-MGB^ | 12934265 | 12934277 |  |  |
|  | RP6 | AGGCCTTTGTTCACATCTCTTCA | 12972165 | 12972187 |  |  |
| ***Airn*-**  **middle** | *Airn*-TQF | GACCAGTTCCGCCCGTTT | 12987545 | 12987562 | 53kb | 17 |
|  | *Airn*TQ | ^FAM-^TACAAGTGATTATTAACTCCACGCCAGCC  TCA^-TAMRA^ | 12987483 | 12987543 |  |  |
|  | *Airn*-TQR | GCAAGACCACAAAATATTGAAAAGAC | 12987483 | 12987508 |  |  |
| ***Airn*-**  **SV1**  **(RP21)** | FP1 | AAGCACAGCACCGCCAGT | 12934245 | 12934262 | 73kb | 17 |
|  | AS | ^FAM-^CCACGCAGACATC^-MGB^ | 12934265 | 12934277 |  |  |
|  | RP21 | CCATGTCCTTTCTTTTCCACTACC | 13007378 | 13007401 |  |  |
| ***Airn*-**  **SV2**  **(RP5)** | FP1 | AAGCACAGCACCGCCAGT | 12934245 | 12934262 | 88kb | 17 |
|  | AS | ^FAM-^CCACGCAGACATC^-MGB^ | 12934265 | 12934277 |  |  |
|  | RP5 | CAAAGGTGCTTGCCTCCAA | 13022721 | 13022739 |  |  |
| ***Airn-***  **end** | *Airn*End-Fwd | GGACTGGCTCAGGCAAGCT | 13032966 | 13032984 | 99kb | 17 |
|  | *Airn*End-P | ^FAM-^CCTGCTCGAGTTGCCATTCCCAGA-^TAMRA^ | 13032938 | 13032961 |  |  |
|  | *Airn*End-Rev | TTCAGTCAAAAATCCAAAACATGT | 13032912 | 13032936 |  |  |
| ***Airn*-**  **SV3**  **(RP4)** | FP1 | AAGCACAGCACCGCCAGT | 12934245 | 12934262 | 118kb | 17 |
|  | AS | FAM-CCACGCAGACATC-MGB | 12934265 | 12934277 |  |  |
|  | RP4 | CAGGACCTCAAGTCAGGAACCT | 13051981 | 13052002 |  |  |
| ***Igf2r*-**  **Ex48** | *Igf2r*-Ex48-F | TCCTACAAGTACTCAAAGGTCAGCA | 7191 | 7215 | -57kb | NM_010515.2 |
|  | *Igf2r*-Ex48-P | ^FAM-^CCAAGACTAGGCAAGGACGGGCAAGA-^TAMRA^ | 7278 | 7303 |  |  |
|  | *Igf2r*-Ex48-R | CGCCTTGGTGGTGATATGG | 7310 | 7328 |  |  |
| **Ex12-**  **q-SNP**  **assay** | WtSeFCG | TGGCCTTGCCCTCCTGC | 1724 | 1740 | -20kb | NM_010515.2 |
|  | MutSeFCG | CTGGCCTTGCCCTCCTGT | 1724 | 1741 |  |  |
|  | GeSeR2 | GCTATGACCTGTCTGTGTTGGCT | 1606 | 1628 |  |  |
| **Cyclo-**  **philinA**  **(Ppia)** | CypA-F | AGGGTTCCTCCTTTCACAGAATT | 178 | 200 | - | BC083076.1 |
|  | CypA-P | ^FAM^-TCCAGGATTCATGTGCCAGGGTGG-^TAMRA^ | 203 | 226 |  |  |
|  | CypA-R | GTGCCATTATGGCGTGTAAAGTC | 228 | 250 |  |  |
| ***Slc22a2*** | *Slc22a2*-F | GGAAAATCGGTGCCAGTCTC | 1023 | 1042 | -131kb | AJ003036 |
|  | *Slc22a2*-P | ^FAM^-CTTCAGAGCCTGACGGCAGATGAGGA-^TAMRA^ | 1044 | 1069 |  |  |
|  | *Slc22a2*-R | AAGGGTTCAATTTCATGCCAGT | 1071 | 1092 |  |  |
| ***Slc22a3*** | *Slc22a3*-F | GAAATGCACGCTCATCCTTATG | 1409 | 1430 | -308kb | AF078750 |
|  | *Slc22a3*-P | ^FAM^-TTGCTTGGTTCACGAGCGCCGT-^TAMRA^ | 1432 | 1453 |  |  |
|  | *Slc22a3*-R | CAGGCGCATGACAAGTCCTT | 1462 | 1481 |  |  |
| **PCR assays** | | | | | | |
| **Ex12-**  **non-q-**  **SNP assay** | Ex12cDNAF | TTCACAGGTGAGGTGGACTG | 1500 | 1519 | -20kb | NM_010515.2 |
|  | Ex12cDNAR | CCGTGCAGTTCTCTCCTTCT | 2021 | 2040 |  |  |
|  |  |  |  |  |  |  |
|  |  |  |  |  |  |  |
| **DNA blot probes** | | | | | | |
| **MEi** |  |  | 12933234 | 12934384 | 0bp | 17 |
| ***Airn*T** |  |  | 12938949 | 12939391 | 4.7kb | 17 |
| **EEi** |  |  | 12961305 | 12966278 | 27kb | 17 |
